# Heading Date QTL in Winter Wheat (*Triticum aestivum* L.) Coincide with Major Developmental Genes *VERNALIZATION1* and *PHOTOPERIOD1*

**DOI:** 10.1371/journal.pone.0154242

**Published:** 2016-05-10

**Authors:** Mohammed Guedira, Mai Xiong, Yuan Feng Hao, Jerry Johnson, Steve Harrison, David Marshall, Gina Brown-Guedira

**Affiliations:** 1 Department of Crop Science, North Carolina State University, Raleigh, North Carolina, 27695, United States of America; 2 Department of Crop and Soil Sciences, University of Georgia, Griffin, Georgia, 30223, United States of America; 3 School of Plant, Environmental and Soil Sciences, Louisiana State University, Baton Rouge, Louisiana 70803, United States of America; 4 USDA-ARS Plant Science Research Unit, Raleigh, North Carolina 27695, United States of America; Institute of Genetics and Developmental Biology, CHINA

## Abstract

In wheat (*Triticum aestivum* L.), time from planting to spike emergence is influenced by genes controlling vernalization requirement and photoperiod response. Characterizing the available genetic diversity of known and novel alleles of *VERNALIZATION1* (*VRN1*) and *PHOTOPERIOD1* (*PPD1*) in winter wheat can inform approaches for breeding climate resilient cultivars. This study identified QTL for heading date (HD) associated with multiple *VRN1* and *PPD1* loci in a population developed from a cross between two early flowering winter wheat cultivars. When the population was grown in the greenhouse after partial vernalization treatment, major heading date QTLs co-located with the *VRN-A1* and *VRN-B1* loci. Copy number variation at the *VRN-A1* locus influenced HD such that RIL having three copies required longer cold exposure to transition to flowering than RIL having two *VRN-A1* copies. Sequencing *vrn-B1* winter alleles of the parents revealed multiple polymorphisms in the first intron that were the basis of mapping a major HD QTL coinciding with *VRN-B1*. A 36 bp deletion in the first intron of *VRN-B1* was associated with earlier HD after partial vernalization in lines having either two or three haploid copies of *VRN-A1*. The *VRN1* loci interacted significantly and influenced time to heading in field experiments in Louisiana, Georgia and North Carolina. The *PPD1* loci were significant determinants of heading date in the fully vernalized treatment in the greenhouse and in all field environments. Heading date QTL were associated with alleles having large deletions in the upstream regions of *PPD-A1* and *PPD-D1* and with copy number variants at the *PPD-B1* locus. The *PPD-D1* locus was determined to have the largest genetic effect, followed by *PPD-A1* and *PPD-B1*. Our results demonstrate that *VRN1* and *PPD1* alleles of varying strength allow fine tuning of flowering time in diverse winter wheat growing environments.

## Introduction

Common wheat (*Triticum aestivum* L.) is the world’s most widely grown crop, accounting for the largest area under cultivation of any single crop [1]. Plasticity of the flowering pathway in wheat is key to adaptation as it allows for regulation of developmental phases in different environments and accounts for the broad geographical range of wheat cultivation. Selection for the most appropriate developmental pattern to suit growing conditions has contributed to global increases in wheat yields [2]. As temperature and moisture regimes in wheat production areas change, selection for genes affecting timing of plant developmental phases will be needed to minimize exposure to harsh conditions and to maintain or increase yields.

Vernalization and photoperiod genes of hexaploid wheat are major regulators of the transition from the vegetative to reproductive growth phase, ensuring that flowering occurs when temperatures are favorable and water is abundant to support growth. Vernalization is defined as the acquisition or acceleration of flowering after exposure to low temperature [3,4]. Wheat has both winter and spring growth habits, with winter wheat requiring exposure to chilling temperatures to transition to reproductive growth. In autumn sown wheat, vernalization sensitivity protects the wheat meristems from cold temperatures during winter by delaying flower initiation [5]. Three groups of genes are reported to regulate vernalization: *VRN1*, *VRN2*, and *VRN3*. *VRN1* is a flowering promoter that remains repressed until it is cold induced [6]. *VRN2* is a strong repressor of flowering and is downregulated by vernalization and short days [7]. *VRN3* accelerates the reproductive development of the apex and is induced by long days [8]. *VRN1* has been reported to be the main flowering signal that promotes the transition of the apex from vegetative to reproductive phase [9–10]. Each wheat genome contains a homoeologus copy of *VRN1* designated *VRN-A1*, *VRN-B1*, and *VRN-D1*, located on the long arms of chromosomes 5A, 5B, and 5D, respectively [11–12]. The presence of a dominant allele in any genome confers spring growth habit, whereas the presence of recessive alleles in the homozygous state across *VRN1* loci confers winter growth habit [13]. Characterization of the *VRN1* genes at the DNA sequence level [6,7,14,15] identified mutations resulting in spring growth habit. At *VRN-A1*, three distinct mutations in the promoter region (*VRN-A1a*) or intron 1 (*VRN-A1b* and *VRN-A1c*) were reported while large deletions within intron 1 of the *VRN-B1* and *VRN-D1* genes resulted in spring growth habit [14].

Variations in winter alleles of *VRN1* have recently been associated with differences in the duration of vernalization required to promote flowering of winter wheat. Díaz et al. [16] identified wheat genotypes having one, two and three *VRN-A1* copies and determined that longer periods of exposure to cold were needed to potentiate flowering for plants having an increased copy number. Chen et al. [17] reported a single-nucleotide polymorphism (SNP) in exon 4 of *vrn-A1* associated with QTL for plant development in winter wheat and designated alleles *vrn-A1a* in early flowering cultivar Jagger and *vrn-A1b* in the cultivar 2174. Li et al. [18] reported subsequently that cultivar 2174 had three *VRN-A1* copies and Jagger had one *VRN-A1* copy. They also demonstrated that a SNP in exon 7 resulting in an Ala^180^ to Val^180^ amino acid mutation in the *vrn-A1* protein decreased its ability to bind with the *Ta*HOX1 protein thought to function in the flowering pathway in winter wheat. The wheat cultivar Claire, having a single *VRN-A1* copy, also carried the exon variants described in Jagger [16]. Differences in vernalization duration requirement associated with *VRN-B1* were reported in a RIL population from the cross AGS 2000× NC-Neuse. A SNP in intron one of *vrn-B1* was the basis of mapping a major QTL for heading date after exposure to four weeks of vernalization treatment, but this variant was not predicted to affect expression of *vrn-B1* [19].

Photoperiod response is also a critical factor affecting the flowering time and adaptation of wheat. In photoperiod sensitive wheat, flowering is accelerated by exposure to long days. Globally, wheat photoperiod insensitivity is widespread in areas where the crop is grown during short days or where wheat maturity is required before the onset of high summer temperatures [20]. Once vernalization requirement of winter wheat is fulfilled, cultivars those are insensitive to photoperiod switch to reproductive growth as spring temperatures increase. Photoperiod response in wheat is mainly controlled by the *PHOTOPERIOD1* (*PPD1)* loci located on the short arms of chromosomes 2A, 2B, and 2D. *PPD1* genes identified in wheat and barley are members of the pseudo response regulator (PRR) family [21,22]. Large deletions upstream of *PPD1* genes in each of the wheat genomes have been reported to decreased sensitivity to photoperiod [22–24]. Copy number variation has also been detected at the *PPD-B1* locus with increased gene numbers leading to earlier flowering [16]. Dominant alleles confer photoperiod insensitivity and are designated by the suffix “a” whereas sensitive alleles are designated by the suffix “b”. The *PPD-D1a* allele having a 2,089 bp deletion in the promoter region [22] is widely considered the most potent allele for photoperiod insensitivity, followed by the *PPD-B1a* and *PPD-A1a* alleles [25,26].

Characterization of *VRN1* and *PPD1* alleles, their relative effects, and interactions is needed to further our understanding of regulation of developmental phases of winter wheat to suit variable environments. The aim of the current study was to investigate the genetic control of heading date in a cross between commercial winter wheat cultivars adapted to the mild winters of the southern U.S. Both parents of our recombinant inbred line (RIL) mapping population, cultivars AGS 2000 and Pioneer variety 26R61 (hereafter referred to as 26R61), are early flowering cultivars. A densely populated linkage map of the population that included allele specific markers for the *VRN1* and *PPD1* loci was used for QTL mapping. The RILs were grown in the greenhouse after different vernalization treatments to identify QTL associated with differences in heading date (HD) associated with vernalization requirement duration. The population was also evaluated at environments in Louisiana, Georgia, and North Carolina to determine the effect of the observed HD QTL in the field and to identify other genes influencing maturity.

## Materials and Methods

Permits and approvals were not required for the work that was conducted on agricultural land owned by North Carolina State University, University of Georgia and Louisiana State University. The land is not privately owned or protected. There are not any protected species sampled. There is not animal husbandry or experimentation.

### Plant Materials

In this study, 175 recombinant inbred lines (RILs) from a cross between soft winter wheat cultivars AGS 2000 (PI612956) and 26R61 (PI612153) described by Hao et al. [12] were evaluated for heading date in both the field and greenhouse. Near isogenic line (NIL) from two F_7_ RILs, AP37 and AP129, for which flowering time variation was observed after partial vernalization treatment were derived as follows. Seeds from each greenhouse plant were harvested separately then sown in single one-meter long rows in the field at Raleigh, NC during fall 2013. At the booting stage, tissue was collected from ten individually tagged plants in each row and DNA was isolated using the QIAGEN DNAeasy Plant Mini Kit (QIAGEN, Valencia, CA, USA). Samples were evaluated with markers for the *PPD-A1*, *PPD-B1*, *PPD-D1*, *VRN-A1* and *VRN-B1* loci (Table 1) and heads from the tagged plants were harvested. Seeds from a single head of plants derived from the same RIL having identical homozygous genotypes at all *PPD1* loci and contrasting genotypes for either the *VRN-A1* or *VRN-B1* locus were selected and seed of NIL pairs increased in the greenhouse.

**Table 1:** Primers for major *VRN1* and *PPD1* genes.

Locus	AGS 2000 allele / Number of copies	26R61 allele / Number of copies	Marker name / Type	Primer names	Sequence (5'-3')	Reference
*VRN-A1*	C/T/T	C/T	TaVrn-A1_Exon4_C/T	Vrn-A1_Exon4_CF1	AGGCATCTCATGGGAGAGGATC	Díaz et al., 2012
	3 copy vrn-A1	2 copy vrn-A1	KASP	Vrn-A1_Exon4_TF2	CAGGCATCTCATGGGAGAGGATT	
				Vrn-A1_Exon4_R	CCAGTTGCTGCAACTCCTTGAGATT	
*VRN-A1*	T	T	TaVrn-A1_Exon7_G/A	Vrn-A1_Exon7_CF1	TGAGTTTGATCTTGCTGCGCCG	Díaz et al., 2012
			KASP	Vrn-A1_Exon7_TF2	CTGAGTTTGATCTTGCTGCGCCA	
				Vrn-A1_Exon7_R	CTTCCCCACAGCTCGTGGAGAA	
*VRN-A1*	GGACC	GGACC	VrnA1_TaGRP2_RIP-3	VrnA1_RIP-3_AL1	CATTGTTGTTGGTATGGACC	This manuscript; Kippes et al. 2015
			KASP	VrnA1_RIP-3_AL2	CATTGTTGTTGGTATCGACT	
				VrnA1_RIP-3_C2	GACTTCATGGTAAAACCCTTTTTGGCATA	
*VRN-B1*	A	C	TaVrn-B1_1752	TaVrnB1_1752_CF1	GGAATGACCGCTGCTTAGTAAATATC	Guedira et al. 2014
			KASP	TaVrnB1_1752_AF2	GGAATGACCGCTGCTTAGTAAATATA	
				TaVrnB1_1752_R	GATTTAGCACCTCAACATACAGGTCT	
*VRN-B1*	437 bp	473 bp	TaVrn-B1_5400	TaVrn-B1_5400F	ACCCACATGATTCTCCACA	This manuscript
			STS	TaVrn-B1_5400R	GCTCTTTTTCCCGCCTGAACT	
*PPD-A1*	*PPD-A1a*.*1*	*PPD-A1b*	TaPpd-A1prodel	Ppd-A1prodel_AL1	TTTCGGTGTTTGACTTCAGGCG	This manuscript; Nishida et al. 2013
			KASP	Ppd-A1prodel_AL2	GCGGCGAGCCGGTTAATCG	
				Ppd-A1prodel_C1	GTGGCGTACTCCCTCCGTTTCTT	
*PPD-B1*	*PPD-B1a*	*PPD-B1b*	TaPpd-B1_S64	TaPpdBJ003F	CGTGAAGAGCTAGCGATGAACA	Díaz et al., 2012
	3-copy Ppd-B1	1-copy Ppd-B1	KASP	TaPpdBJ003R	TGGGCACGTTAACACACCTTT	
*PPD-D1*	*PPD-D1b*	*PPD-D1a*	wMAS000024	TaPpdDD001RI	CAAGGAAGTATGAGCAGCGGTT	www.cerealsdb.uk.net/cerealgenomics/CerealsDB/kasp_download.php?URL=
			KASP	TaPpdDD001RD	AAGAGGAAACATGTTGGGGTCC	
				TaPpdDD001FL	GCCTCCCACTACACTGGGC	

Allele specific primers used to determine alleles of genes affecting vernalization and photoperiod response present in wheat cultivars AGS 2000 and 26R61. Competitive allele specific PCR (KASP) assays do not include tail sequences.

### Phenotypic Evaluation

The effect of vernalization on flowering was evaluated on the RIL population and the parents in the greenhouse after being vernalized for 8 weeks (8W), 4 weeks (4W) or 2 weeks (2W) at 4°C. The vernalization process was performed by germinating seeds in paper towels modified with a wick that was partially immersed in distilled water to maintain saturation. After vernalization, seedlings were transplanted into black plastic Containers (Stuewe & Sons, Tangent, Oregon USA) (D40: Volume 0.7L, 6.5 cm in diameter and 25 cm depth) containing 1:1 Metro Mix 2 and soil and 2 g of slow release fertilizer (Multicote 14-14-16, N-P-K) was incorporated into the soil. Six plants per entry/treatment combination were grown in a completely randomized design in a greenhouse with 16 hrs photoperiod and 20/15°C day/night temperature. Heading date was recorded when plants were at Zadocks 59 growth stage [27].

Germinated seed of F_7_ derived NIL pairs and parents were vernalized for 0, 2, 4, 6 and 8 weeks. Ten seedlings of each line were then transplanted into black plastic containers and grown in the greenhouse as described above. Heading date was recorded when the first head completely emerged at Zadocks 59 growth stage. ANOVA was conducted to test for heading time differences between the NIL pairs and parent lines.

Heading date was evaluated on the RILs, along with parents, grown at field locations including Raleigh, NC (Latitude: 35.73°; Longitude: -78.68°; Elevation: 116.5 m) in harvest years 2012, 2013 and 2014, and Plains, GA (Latitude: 32.05°; Longitude: -84.37°; Elevation: 150 m) and Baton Rouge, LA (Latitude: 30.54°; Longitude: -91.15°; Elevation: 20 m) in 2012. Three grams of seed of each RIL and parents were planted in 1.5 m rows in a randomized complete block design with three replications. Heading date was recorded as day of year in all environments when 50% of the plants in a plot were at Zadocks 59 growth stage [27].

### Genotyping

The AGS 2000 x 26R61 RIL population was previously genotyped using SSR markers and DArT genetic markers as described by Hao et al. [28–30]. In this study, single nucleotide polymorphism (SNP) markers were also evaluated on the population using the Illumina iSelect array for wheat described by Cavanagh et al. [31]. A total 2833 polymorphic markers were identified.

Genotypes of parents, RILs and NILs for major vernalization and photoperiod loci were determined using competitive allele-specific PCR (KASP) assays (Table 1). KASP assays for different winter alleles of the *VRN-A1* and *VRN-B1* loci and for detection of insensitive alleles of the *PPD-B1* and *PPD-D1* loci were performed as previously described [16,19]. A new KASP assay was designed for *VRN-A1* that targeted the recently reported *TaGRP2* RIP-3 binding site mutation [32]. A new assay targeting the large insertion/deletion polymorphism in the promoter of *PPD-A1* [24] was also developed (Table 1). KASP reactions were setup in a 5 μL reaction containing 2.5 μL 2X KASP Master Mix, 0.07 μL 72X Assay mix (containing allele specific and common primers), and 2.5 μL of gDNA (~ 30–50 ng/μL). PCR cycling conditions were programmed according to manufacturer’s instructions (LGC Genomics, Middlesex, UK) and endpoint genotyping was conducted using a Pherastar plate reader (BMG Labtech, Otenberg, Germany) and the software Klustercaller (LGC Genomics, Middlesex, UK).

### Map construction and QTL analyses

A chi-square test was performed to test for goodness-of-fit of markers to an expected ratio of 1:1. Linkage mapping was performed using QTL ICIMapping V4.0 [33] (http://www.isbreeding.net/). The BIN tool was implemented to identify and remove redundant markers in the dataset and also remove markers with high levels of missing data. Linkage groups were created using the “Grouping” tool and were selected based on minimum LOD score of 4. Marker distances in cM were calculated using the Kosambi function and the “nearest neighbor algorithm” option. Linkage group identity and order were assigned based on evaluation of similarities to published linkage maps available in GrainGenes (http://wheat.pw.usda.gov) and the SNP map of Cavanagh et al. [31]. Retained and mapped markers were used for QTL analyses. Single environment QTL analysis was conducted for each vernalization treatment by the method of inclusive composite interval mapping (ICIM) in QTL IciMapping ver. 4.0. LOD thresholds for QTL detection were determined by 1000 permutations QTLs with epistatic effects were detected by selecting ICIM-EPI with a probability value for entering variables (PIN) of 0.0001. The default LOD of 3.0 for ICIM-EPI was used to detect epistatic QTLs. Epistatic effects of significant QTL were calculated with the software R version 1.32–6 [34]. An additional analysis was performed by grouping lines into classes according to their genotypic status at the VRN1 and PPD1 loci. An analysis of variance fitting genotypic class as a fixed main effect was done for each environment separately using Proc GLM in SAS 9.3 (SAS Institute, Inc., Cary, NC). The average least difference comparing any two classes was computed for each environment.

### *VRN-B1* Sequencing

Genomic DNA of AGS 2000 and 26R61 were used to amplify the 13 kb *VRN-B1* gene by parts. Five genome specific PCR primer pairs and 25 sequencing primers were designed from the *vrn-B1* Triple Dirk C (AY747604) sequence (S1 and S2 Tables). PCR amplification was done in a 50 μL volume containing 34.05 μL distilled water, 5 μL of 10x high fidelity buffer (Invitrogen, Grand Island, NY), 6 μL dNTPs (2.5 mM), 2.0 μL of premixed forward and reverse primers (10 μM), 0.75 μL dimethyl sulfoxide, 2 μL MgSO_4_ (50 mM), 0.20 μL Platinum Taq DNA Polymerase (Invitrogen) and 400 ng of gDNA. The PCR program was: 95°C for 3 min, followed by 94°C for 30 s, annealing at 65°C for 30 s, and extension at 68°C for 6 min (6 kb amplicon), 3 min (2.5 kb and 1.75 kb amplicons), or 1 min (860 bp amplicon) depending on the expected amplicon size. The annealing temperature was reduced by 1°C for every additional cycle until 55°C. Finally, 25 cycles were done at 94°C for 30 s, 55°C for 30 s, 68°C for 6 min, 3 min, or 1 min depending on the expected amplicon size. PCR amplicons were then purified using the QIAquick PCR purification kit (QIAGEN, Valencia, CA, USA) to remove residue PCR primers. The purified PCR amplicons were either sequenced directly or cloned into cloning vector *pCR 2*.*1 TOPO* (Invitrogen) for Sanger sequencing. Sequences from the cultivars were assembled into individual contigs using the CAP3 sequence assembly program. The assembled contigs were then blasted against the Triple Dirk C v*rn-A1 (*AY747600), *vrn-B1 (*AY747604), and *vrn-D1* (AY747606) genes to verify homology to the *vrn-B1* gene sequence. Variant identification was conducted by aligning the assembled AGS 2000 (KR816809) and P26R61 (KR816810) *vrn-B1* gene sequences in Clustal.

A new co-dominant PCR assay was developed using genome specific primer pairs to assay the presence/absence of the 36 bp deletion identified in the first intron of the AGS 2000 *vrn-B1* allele (Table 1). PCR was setup in a 10 μL reaction volume containing 7.45 μL water, 1.2 μL 10x Buffer (New England Biolabs, Ipswhich, MA), 0.96 μL of 2.5 mM dNTPs, 0.30 μL of 10 μM premixed primers (forward and reverse), and 0.09 μL Taq Polymerase (New England Biolabs). PCR amplification program was 95°C for 1 min, followed by 30 cycles of denaturing at 94°C for 30 s, annealing at 55°C for 30 s, and extension at 68°C for 1 min. Fragments were resolved using an ABI 3730xl (Life Technologies, Carlsbad, CA).

### Copy Number Assays

Vernalization and photoperiod gene copy number estimates were determined using *VRN-A1* and *PPD-B1* TaqMan^®^ assays previously described by Díaz et al.[16], and a *VRN-B1* TaqMan^®^ assay developed in this study (forward primer: 5’-*CAGCATTCATCCAGCGGCAT*-3’, reverse primer: 5’-*CTTCAGCCGTTGATGTGGCTA*-3’, probe primer: Fam\5’-*CAGAGGATGCGGCAGTGCAG*-3’\TAMARA). The *VRN-B1* TaqMan^®^ assay was designed around an intron and exon junction at exon 8 that presented sufficient sequence variation for designing a genome specific assay. The *VRN-B1* TaqMan^®^ assay genome specificity and copy number differences were confirmed using genomic DNA isolated from Chinese Spring nullisomic-ditelosomic (N5B-D5A + Dt5AL) and nullisomic-tetrasomic (N5D-T5B) lines. The cultivars Claire, Malacca, and Hereward were used as controls for determining *VRN-A1* copy number. Cultivars Chinese Spring and Alchemy were used as controls to evaluate *PPD-B1* copy number differences.

Copy number assays were conducted in 10 μL reaction volume consisting of 5 μL 5X TaqMan Universal Master Mix II (Invitrogen), 1 μL of premixed gene specific primers (3 μM forward, 3 μM reverse, and 2 μM probe), 1 μL of premixed *TaCO* internal control primes (2 μM forward, 2 μM reverse, and 1 μM probe), 1 μL diH_2_0, and 2 μL standardized DNA. Quantitative PCR was conducted in a Roche LightCycler LC 480 programmed to start heating at 95°C for 10 min follow by 40 cycles of denaturing at 95°C for 15 s and 60°C annealing and extension for 1 min. PCR cycle threshold (Ct) was determined for the gene of interest and the single copy gene *TaCO* was used for normalization. Copy number was estimated by taking the target and control gene ratio 2^-(Target Ct—Control Ct)^ for at least ten technical replicates for the parent and control lines and four technical replicates for the RILs. ANOVA analysis was conducted to determine copy number difference between the parents and control lines.

## Results

### Genotypes at *PPD1* and *VRN1* loci

**Fig 1:**
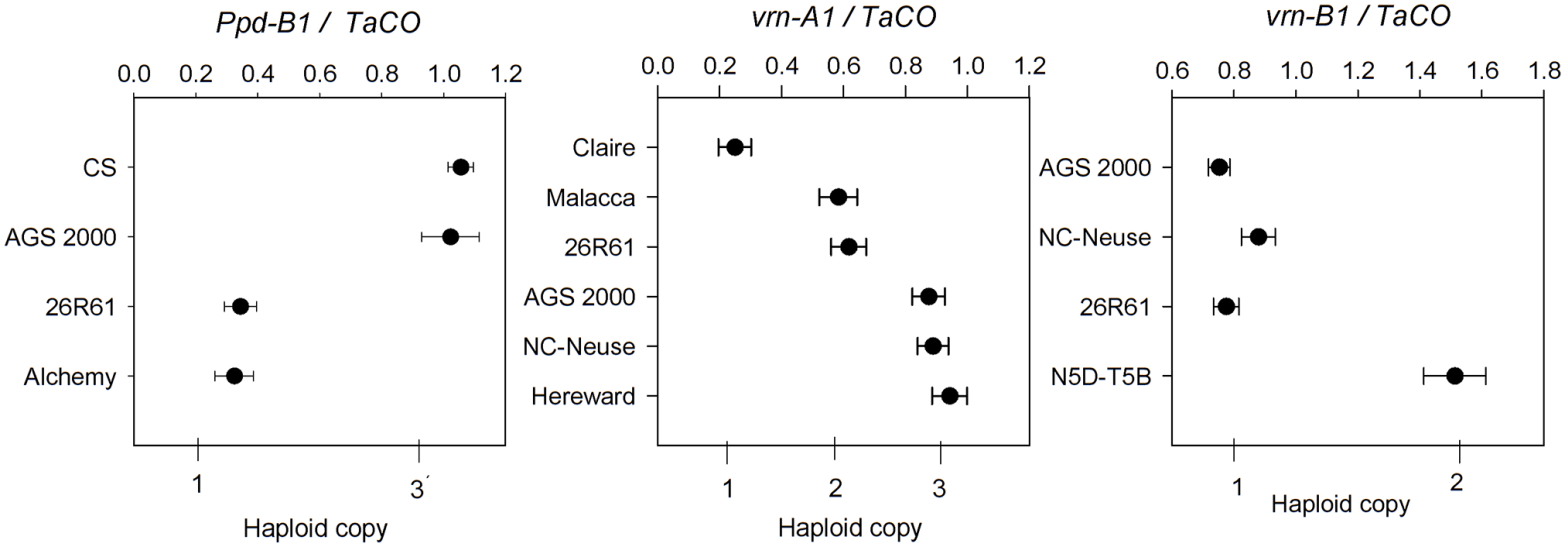
Copy number variation for *PPD-B1*, *VRN-A1* and *VRN-B1*. TaqMan^®^ estimates of haploid copy number in relation to internal positive control signal ratio (TaCO) for *PPD-B1*, *VRN-A1* and *VRN-B1*. CS: Chinese Spring; N5D-T5B: Chinese Spring nullisomic 5D-tetrasomic 5B. Means and standard deviations of ten measurements are shown.

Marker analysis determined that AGS 2000 and 26R61 differed at the vernalization and photoperiod loci evaluated (Table 1). Cultivar 26R61 has photoperiod sensitive *PPD-A1b* and insensitive *PPD-D1a* alleles. In contrast, AGS 2000 has the photoperiod insensitive *PPD-A1a*.*1* and sensitive *PPD-D1b* alleles. Differences were also observed for the *PPD-B1* locus. KASP assay TAPpdBJ003 indicated that AGS 2000 has *PPD-B1a* characterized by the intercopy junction observed in cultivars Sonora/Timstein with multiple copies of the *PPD-B1* gene and 26R61 carries the sensitive *PPD-B1b* allele. Analysis of *PPD-B1* copy number showed that AGS 2000 had three haploid copies and 26R61 had one haploid copy (Fig 1). The TAPpdBJ003 KASP assay was used to genotype the mapping population and NILs.

Although none of the reported mutations in wheat *VRN1* genes leading to spring growth habit were observed in either parent, polymorphism between AGS 2000 and 26R61 was observed for the winter alleles of vernalization loci *VRN-A1* and *VRN-B1* (Table 1). Polymorphism was observed for KASP marker designed around a SNP in exon 4 of *vrn-A1* reported by Díaz et al. [16]. No polymorphism was detected at *vrn-A1* exon 7 previously associated with reduced vernalization or the intron 1 *TaGRP2* RIP-3 binding site. TaqMan^®^ assays showed that 26R61 had a haploid copy number of two and cultivars AGS 2000 and NC-Neuse were estimated to have a *VRN-A1* haploid copy number of three (Fig 1). The control lines Claire, Malacca, and Hereward were determined to have *VRN-A1* haploid copy numbers of one, two and three, respectively, as previously demonstrated [16]. When the RILs were evaluated for *VRN-A1* gene copy, 118 RILs were estimated to have a haploid copy of two and 57 RILs were estimated to have a haploid copy of three. Genotyping of the population with the exon 4 KASP assay was in agreement and also indicated an excess of the *vrn-A1* allele from 26R61 (2 haploid copies).

Evaluation with the *TaVrnB1_1752* SNP in the first intron of *VRN-B1* reported to be associated with differences in vernalization duration requirement [19] revealed polymorphism between AGS 2000 and 26R61. To determine if *VRN-B1* copy number variation was present, a TaqMan^®^ assay specific to *VRN-B1* was developed. The Chinese Spring nullisomic5D-tetrasomic5B line used as a control was estimated to have two haploid copies and was different (*p < 0*.*0001*) from AGS 2000, 26R61, and NC-Neuse that were all estimated to have one haploid copy (Fig 1).

To identify polymorphisms in the winter *vrn-B1* alleles, sequenced amplicons from AGS 2000 and 26R61 were assembled into individual contigs spanning 12,858 bp and 12,902 bp respectively. The assembled genes share 99% homology to the winter *vrn-B1* (AY747604) and less than 94% homology to *vrn-A1* (AY747600) and *vrn-D1* (AY747606). Alignments detected SNP upstream of the promoter regions, in intron 5, and the 3’ UTR region. The most extensive variation was observed within the first intron having 35 SNPs and 4 INDELs, including a 36 bp deletion in AGS 2000 that spans from nucleotide interval positions 5400 bp to 5436 bp relative to the Triple Dirk C *vrn-B1* (AY747604) sequence (S1 Fig). No sequence variation was detected between exonic regions or the remaining introns. A new marker TaVrn-B1_5400 was developed around the 36 bp deletion in intron 1 that co-segregated with the TaVrnB1_1752 SNP assay in the RIL population (Table 1). Observed segregation for the two markers at the *Vrn-B1* locus did not differ from the 1:1 ratio expected. The allele carrying the 36 bp deletion in AGS 2000 is hereafter referred to as *vrn-B1_del* and the intact allele from 26R61 as *vrn-B1_non-del*.

### Linkage map

A more dense genetic linkage map was constructed for the AGS 2000 x 26R61 RIL population that included 791 iSelect SNP, 419 DArT, 188 SSR, as well as markers specific for photoperiod and vernalization genes (S3 Table). The map consists of 25 linkage groups assigned to all 21 wheat chromosomes and spanned 3124.3 cM with 1286.3 cM, 1161.9 cM and 673.2 cM in the A, B, and D genomes, respectively (S4 Table). Markers for the *PPD-A1*, *PPD-B1* and *PPD-D1* loci were located at 62.2 cM, 94.6 cM and 9.6 cM on chromosomes 2A, 2B and 2D, respectively. The *VRN-B1* locus was located at 142.8 cM on chromosome 5B. Chromosomes 1D, 5A, 7A and 7D each consisted of two unlinked groups and *VRN-A1* was located at 16.9 cM on linkage group 5A.1.

### Effect of vernalization treatments on heading date

**Fig 2:**
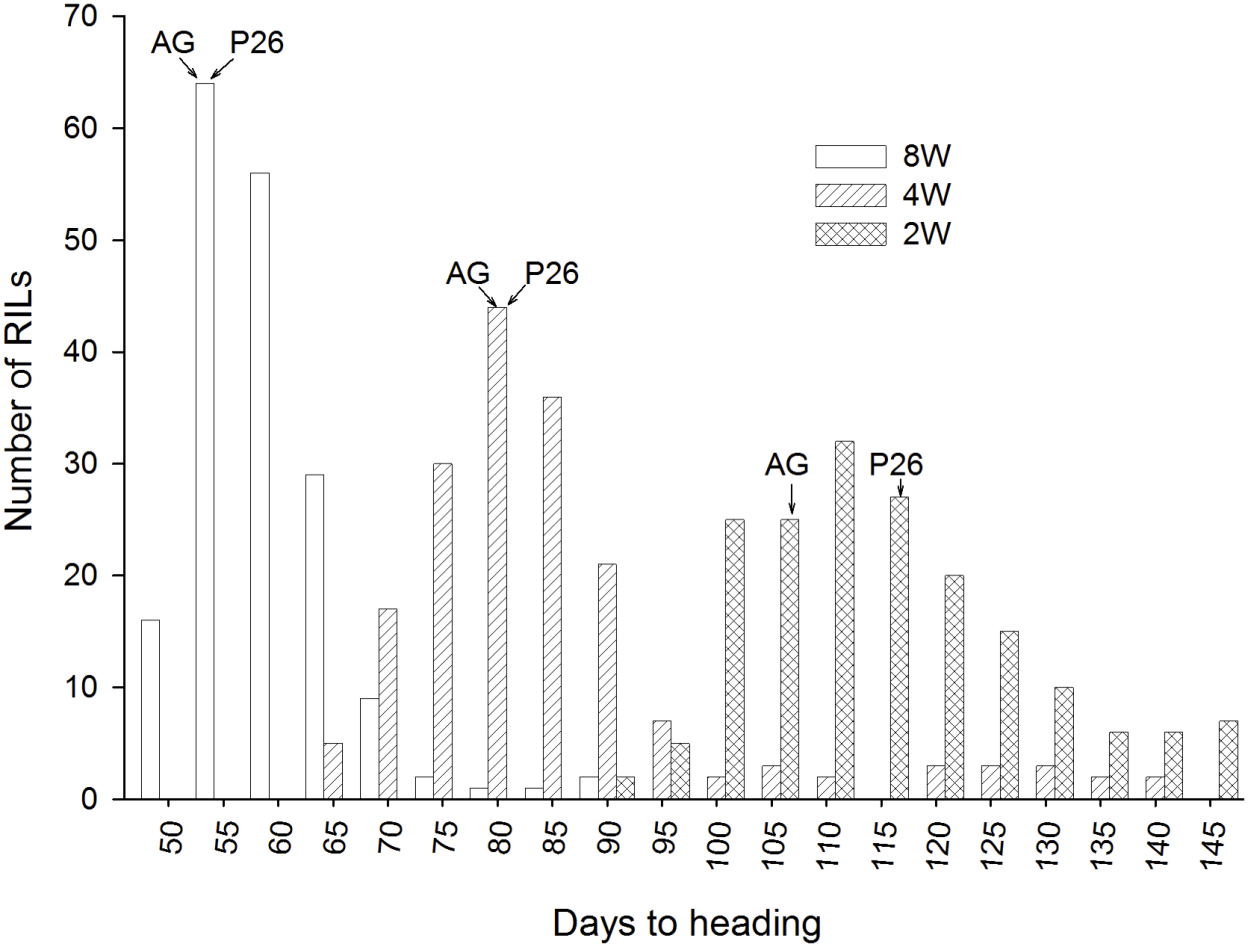
Greenhouse heading dates. Frequency distribution for heading dates of recombinant inbred lines from a cross between AGS 2000 (AG) and 26R61 (P26) after 8 weeks; 8W, 4 weeks; 4W, and 2 weeks; 2W, of vernalization treatment.

Winter wheat cultivars AGS 2000 and 26R61 responded to the vernalization duration treatments used in this study. Heading date decreased significantly for both cultivars as the length of the vernalization treatment increased from 2W to 8W. Significant differences in HD between AGS 2000 and 26R61 were not observed after 8W and 4W of vernalization. However, after 2W vernalization 26R61 headed 10 days later than AGS 2000 (Fig 2). Although HD of the AGS 2000 and 26R61 parents were similar in the 4W and 8W vernalization treatment, a broad range in HD was observed in the RIL population in all treatments (Fig 2). The greatest range in HD for the population was observed after 4W of vernalization (63 to 137 days) followed by the 2W treatment (87 to 144 days). Analysis of variance indicated significant effects of genotypes, treatments, and genotype x treatment interaction (*p <0*.*001*). QTL analyses were therefore done separately for each treatment.

### QTL for heading date in the vernalization treatments

**Fig 3:**
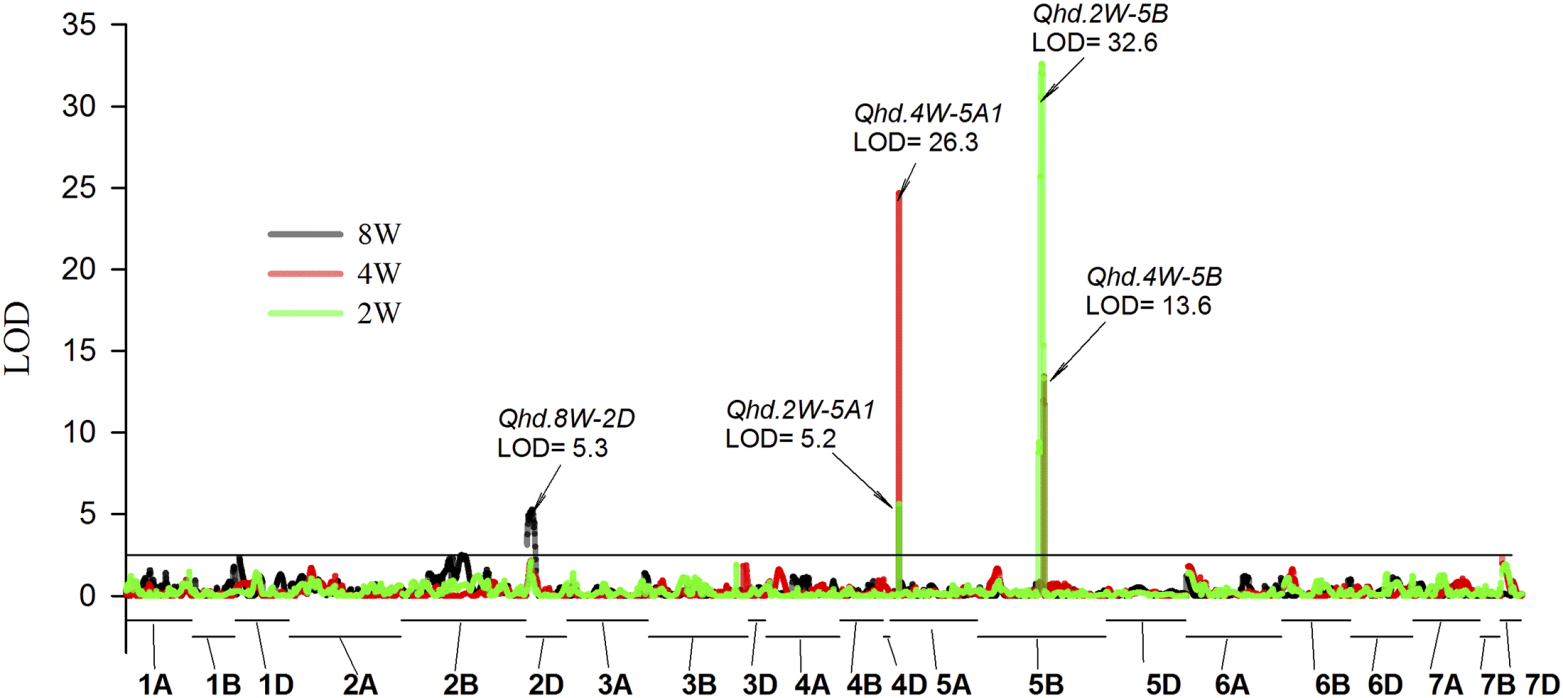
Greenhouse Manhattan plot. Genome scan of logarithm of the odds (LOD) scores for heading dates in AGS 2000 × 26R61 recombinant inbred lines in greenhouse experiments after 8 weeks; 8W, 4 weeks; 4W, and 2 weeks; 2W of vernalization treatment.

The QTL of largest effect in the fully vernalized treatment was associated with the *PPD-D1* locus on chromosome 2D (*Qhd*.*8W-2D*) that explained 15.3% of the variation (Fig 3). The *PPD-D1b* allele from AGS 2000 contributed to later flowering with an additive effect of 2.5 days (Table 2). QTL with large effect on HD in the partial vernalization treatments were identified in chromosomes 5A and 5B (Fig 3). In both treatments, the QTL peaks *Qhd*.*4W-5A*.*1*, *Qhd*.*2W-5A*.*1* and *Qhd*.*4W-5B*, *Qhd*.*2W-5B* were centered over the KASP markers for the *VRN-A1* and *VRN-B1* loci, respectively. After four weeks of cold treatment, the highly significant QTL for HD associated with *VRN-A1* (LOD = 26.3) explained 43.2% of the variation (Table 2). The effect of *VRN-B1* in the 4W treatment was also highly significant (LOD = 13.6) and explained 18.3% of the variation. *VRN-B1* was the most significant locus associated with HD after two weeks of vernalization (LOD = 32.6) and explained 57.9% of the variation. The QTL associated with *VRN-A1* in the 2W vernalization treatment explained 5.9% of the variation. The additive effects of the AGS 2000 *vrn-B1_del* allele were -6.6 and -9.8 days in the 4W and 2W treatments, respectively. In the case of *VRN-A1*, the 26R61 allele (*vrn-A1_2-copy*) contributed to early heading. The additive effects of the AGS 2000 *vrn-A1_3-copy* allele were 10.4 and 3.3 days in the 4W and 2W treatments, respectively.

**Table 2:** Major QTLs for heading date in the greenhouse experiment.

Locus	Linkage group	Position (cM)	Vernalization Treatment	LOD	PVE(%)	Additive effect	Early HD allele
*PPD-D1*	2D	9.6	8W	5.3	15.3	2.5	*PPD-D1a*
*VRN-A1*	5A1	16.9	4W	26.3	43.2	10.4	*vrn-A1_2copy*
			2W	5.2	5.9	3.3	*vrn-A1_2copy*
*VRN-B1*	5B	142.8	4W	13.6	18.3	-6.5	*vrn-B1_del*
			2W	32.6	57.9	-9.8	*vrn-B1_del*
VRN-A1*VRN-B1			4W	17.2	17.1	-6.6	
			2W	6.3	7.3	-3.7	

LOD scores, phenotypic variation explained (PVE), additive effect and early HD allele of the AGS 2000 alleles for the most significant markers in the QTL regions for heading date in the AGS 2000 × 26R61 RIL population grown in the greenhouse after different vernalization treatment; 8 weeks; 8W, 4 weeks; 4W, and 2 weeks; 2W. Unit of additive effect for HD is days.

Epistatic interactions between *VRN-A1* and *VRN-B1* were significant in both partial vernalization treatments and explained an additional 17.1% and 7.3% of the variation in the 4W and 2W vernalization treatments, respectively. The interaction was such that when partially vernalized, RILs having the *vrn-A1_3-copy* allele from AGS2000 and the intact *vrn-B1* allele from 26R61 (*vrn-B1_non-del*) headed later than lines having either or both of the alleles associated with short vernalization requirement (Table 3). Significant differences were observed between all four genotypic classes after 4W of vernalization, with RIL having both of the weak winter alleles (*vrn-A1_2-copy* and *vrn-B1_del)* heading earliest, followed by RIL with the *vrn-B1_non-del* and *vrn-A1_2-copy* combination. After 2W of vernalization, the earliest HD was observed for lines having the weak *vrn-B1_del* winter allele, regardless of *VRN-A1* copy number (Table 3).

**Table 3:** Effect of *VRN1* allele combinations on heading date in the greenhouse and field conditions.

	*VRN1* allele combination				LSD^a^
	*vrn-A1_2-copy*, *vrn-B1_non-del* (n = 61)	*vrn-A1_2-copy*, *vrn-B1_del* (n = 57)	*vrn-A1_3-copy*, *vrn-B1_non-del* (n = 23)	*vrn-A1_3-copy*, *vrn-B1_del* (n = 34)	
Vernalization Treatment	Number of days after transplanting				
8W	56.3	58.1	59.2	58.1	2.6
4W	79.8	74	113	85	4.1
2W	118.2	103.9	131	104.5	3.8
Environments	Day of year				
LA12	78.1	72.3	88	74.3	2
GA12	82.2	80.6	92.2	81.1	2
NC12	90.7	89.3	96.9	90.9	1.8
NC13	108.6	108.9	113.1	108.3	1.4
NC14	116.4	116.4	118.9	116.9	1

Mean heading dates of AGS 2000 × 26R61 recombinant inbred lines (RILs) having different *VRN1* allele combinations when grown in field conditions and the greenhouse under different vernalization treatments; 8 weeks; 8W, 4 weeks; 4W, 2 weeks; 2W. Environments are Baton Rouge, LA, 2012; LA12, Plains, GA, 2012; GA12 Raleigh, NC, 2012; NC12, 2013; NC13, 2014; NC14.

^a^ LSD = Least Significant Difference (*p*<0.05).

### Effect of *VRN-A1* and *VRN-B1* on flowering of near-isogenic lines

**Fig 4:**
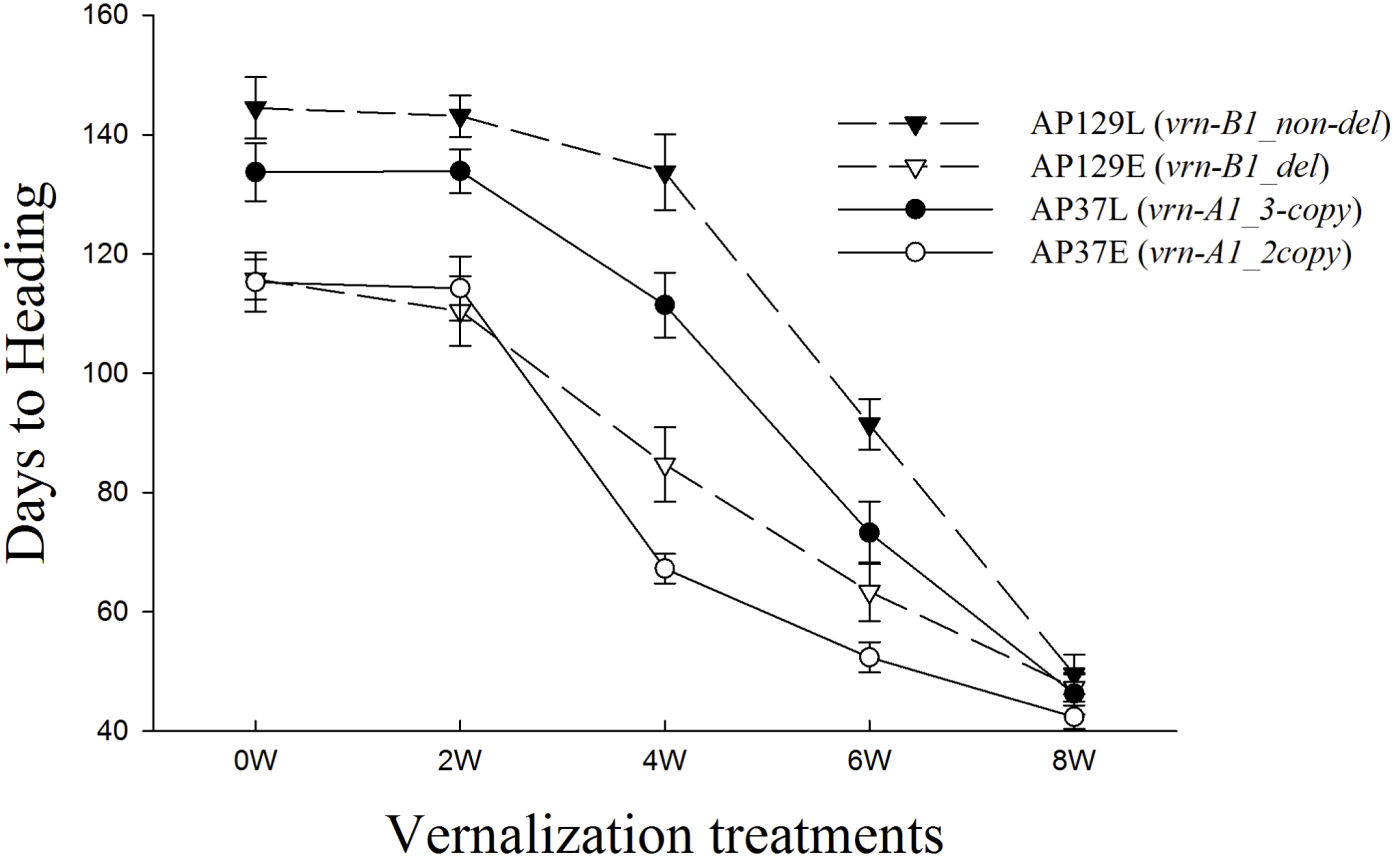
Effect of vernalization treatment on days to heading of near isogenic lines (NILs). NILs originate from recombinant inbred lines AP129 and AP37. NILs AP129 have *PPD-A1a*, *PPD-B1a*, *PPD-D1a*, *vrn-A1_3-copy* and differ for *VRN-B1* alleles. AP129L has the *vrn-B1_non-del* allele while AP129E has the *vrn-B1_del* allele. AP37E and AP37L have *PPD-A1a*, *PPD-B1b*, *PPD-D1a*, *vrn-B1_non-del* and differ for *VRN-A1* alleles. AP37E has *vrn-A1_2-copy* while 37L has *vrn-A1_3-copy*.

The NIL pairs allowed the direct comparisons between sister lines with contrasting *VRN-B1* or *VRN-A1* variants. Near isogenic line pairs AP129E and AP129L were developed to have fixed *PPD1* loci and three copies of *VRN-A1* but differed for *VRN-B1* alleles. Likewise NIL pair AP37E and AP37L had contrasting *VRN-A1* copy number but were fixed for the *PPD1* loci and had the *vrn-B1_ non-del* allele. Heading date was not different when the NIL pairs were fully vernalized for eight weeks (Fig 4). When plants received no vernalization (0W) or partial vernalization for 2W, 4W, and 6W, days to heading was significantly reduced in line AP129E that had the short vernalizing *vrn-B1_del* variant and in line AP37E which had the *vrn-A1_2-copy* variant. Differences in HD were greatest for the *VRN-B1* NIL pair AP129 in all partial vernalization treatments. However, the largest decrease in HD was observed for NIL AP37E after four weeks of vernalization (Fig 4). No significant HD difference was detected between the parent lines at 0W, 4W, and 8W vernalization (data not shown). AGS 2000 headed earlier than 26R61 in the 2W treatment, consistent with observations in conjunction with the RIL population. However, AGS 2000 was nine days later than 26R61 in the 6W vernalization treatment.

### QTL for heading date in the field

**Fig 5:**
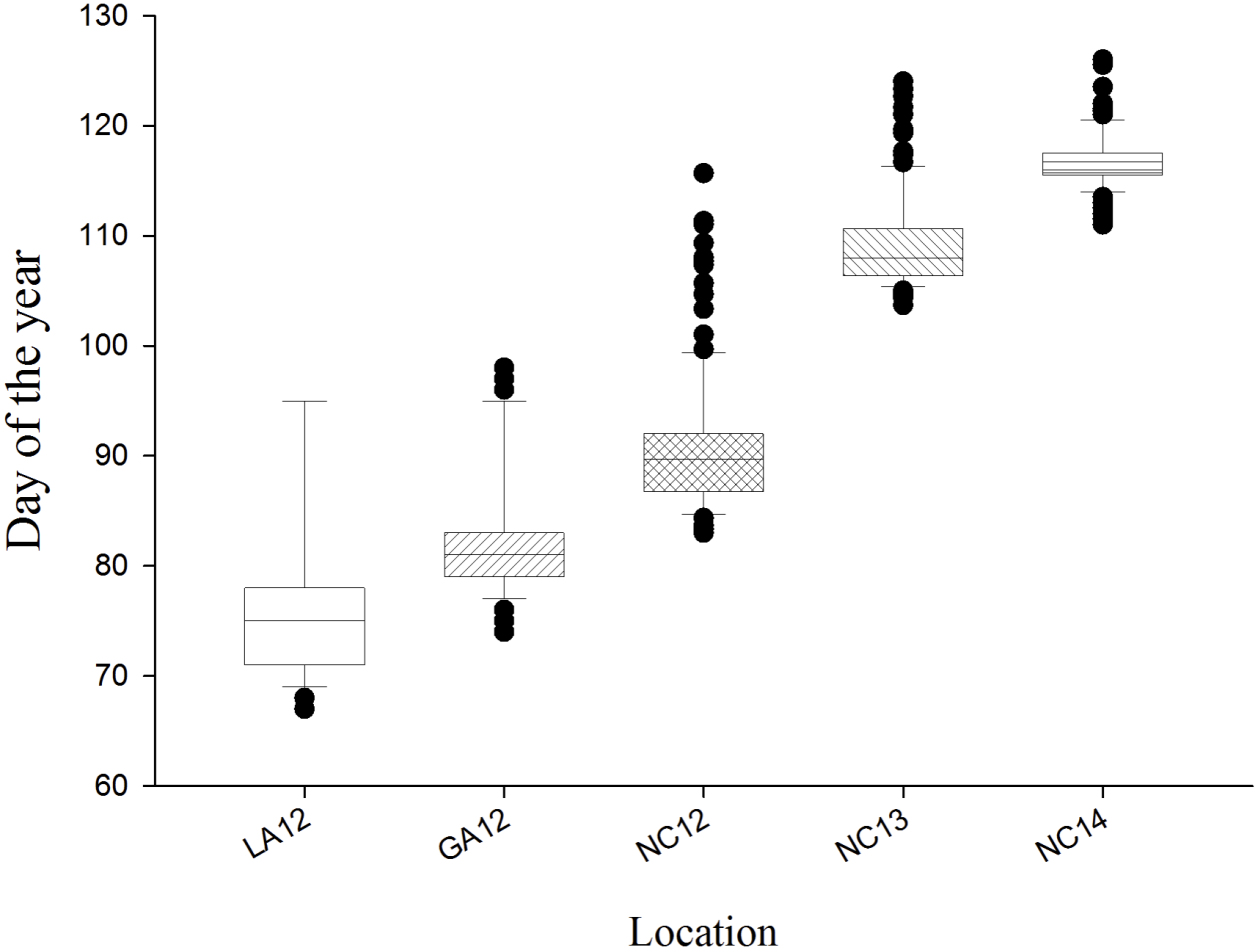
Field heading dates. Box plots of the distribution of heading dates in AGS 2000 × 26R61 in recombinant inbred lines in field experiments. Baton Rouge, LA, 2012; LA12, Plains, GA, 2012; GA12, Raleigh, NC, 2012; NC12, 2013; NC13, 2014; NC14.

When the mapping population and parents were evaluated at field locations in Louisiana (LA12), Georgia (GA12) and North Carolina (NC12, NC13, NC14), significant heading date variation was observed for genotypes, environments and genotype × environment interactions (*p < 0*.*001*). Mean and range of HD in the RILs varied across years and locations (Fig 5). Mean HD was earliest at LA12 (March 16) followed by GA12 (March 23) and NC12 (April 2). Early spring temperatures at Raleigh, NC were warmer in 2012 than in 2013 and 2014 and growing degree days after January 1 accumulated more slowly in the latter two years (S2 Fig). Later mean HDs were observed for the population at the NC13 (April 20) and NC14 (April 28) environments that also had the least variation for HD (Fig 5). The widest range in HD was at NC12 (33 days), followed by a 23 day range in both GA12 and LA12. However, the experiments at LA12 and GA12 ended at 95 and 98 days, respectively. Seventeen RILs at LA12 and 10 RILs at GA12 that were extremely late and had not yet flowered were given a HD as the last day of the experiment.

Despite variation across years and locations, stable HD QTL were associated with *VRN1* and *PPD1* genes. Significant QTL associated with *PPD-A1* and *PPD-D1* were detected in each individual environment. *PPD-D1* explained from 14.5% to 22.1% of the variation in HD and additive effect of the *PPD-D1b* photoperiod sensitive allele ranged from 1.1 days (NC14) to 3.5 days (NC12). Similarly, the QTL associated with *PPD-A1* explained from 6.2% to 20.7% of the variation in HD in individual environments. Additive effect of the *PPD-A1a*.*1* insensitive allele ranged from -0.7 days (NC14) to -3.3 days (LA12). A QTL associated with *PPD-B1* was identified in three of the five environments, LA12, NC12 and NC13. This QTL explained from 3.6% to 5.8% of variation in heading and additive effect of the *PPD-B1a* insensitive allele in these environments ranged from -1.0 day (NC13) to -1.6 days (NC12) (Table 4).

**Table 4:** Major QTLs for heading date in the field experiments.

Locus	Linkage group	Position (cM)	Range of LOD scores		Range of variation explained (%)		Range of additive effects (days)		Early HD allele	Environments
			Min	Max	Min	Max	Min	Max		
*PPD-A1*	2A	62.2	3.3	12.9	6.2	20.7	-0.7	-3.3	*PPD-A1a*	NC12, LA12, NC13, GA12, NC14.
*PPD-B1*	2B	94.6	3.1	3.3	3.6	5.8	-1.0	-1.6	*PPD-B1a*	NC12, NC13, LA12.
*PPD-D1*	2D	9.6	7.4	15.5	14.5	22.1	1.1	3.5	*PPD-D1a*	NC12, LA12, NC13, GA12, NC14.
*VRN-A1*	5A1	16.9	2.6	9.2	6.1	12.3	0.7	2.9	*vrn-A1_2-copy*	LA12, GA12, NC14, NC12, NC13.
*VRN-B1*	5B	142.8	3.7	23.1	6.1	25.8	-1.6	-4.7	*vrn-B1_del*	NC12, LA12, GA12
VRN-A1*VRN-B1	5A1*5B		5.6	6.5	12.2	17.4	-2.3	-2.6		LA12, GA12.

Range of LOD scores, phenotype variation explained (PVE), and additive effect of the AGS 2000 alleles for the most significant markers in the QTL regions for heading date (HD) in the AGS 2000 × 26R61 RIL population grown in Baton Rouge, LA, 2012; LA12, Plains, GA, 2012; GA12 Raleigh, NC, 2012; NC12, 2013; NC13, 2014; NC14. Unit for additive effect for HD is days. QTL effect was detected in the individual environments indicated. Environments are listed in order from largest to smallest amount of variation explained by each locus.

Significant QTL at the *VRN-A1* and *VRN-B1* loci were also detected. The *VRN-A1* QTL was detected in all environments and largest effects were observed at LA12 and GA12 where the locus explained 12.3% and 11.7% of variation, respectively. Additive effects of the *vrn-A1_3-copy* allele were 2.8 and 2.1 days at LA12 and GA12, respectively. The *VRN-A1* allele effects on HD were smaller in all three years at Raleigh, NC (0.8 to 1.7 days) where it accounted for a smaller amount of HD variation (4.9 to 8.1%). The *VRN-B1* locus was highly significant at the NC12 (LOD 3.7), GA12 (LOD 8.4) and LA12 (LOD 23.1) environments where it explained 6.1 to 25.8% of the variation in HD, respectively. Additive effect of the AGS 2000 *vrn-B1_del* allele was greatest at LA12 (-4.7 days) followed by GA12 (-2.6 days). Significant epistatic interactions between *VRN-A1* and *VRN-B1* were detected at the LA12 and GA12 environments (Table 4).

### Single and Multi-Locus Genotype Heading Dates

Mean HD comparisons were done for single and multi-locus genotypes of RIL having combinations of *VRN1* genes (4 classes) or *PPD1* genes (8 classes). Analyses of multi-locus genotypes for *VRN1* and *PPD1* together was not done as the 32 genotypic classes were each represented fewer than 10 times in the population.

Mean HD of RIL classified as possessing alleles for either photoperiod sensitivity or insensitivity at each of the *PPD1* loci were compared to determine the relative effect of the photoperiod alleles individually and in combinations. Mean differences between groups of genotypes having contrasting *PPD1* alleles were generally in agreement significant QTLs identified. However, significant differences (*p<0*.*01*) were observed for all environments between allelic classes at the *PPD-B1* locus although a significant QTL associated with *PPD-B1* was only detected in three of the five environments (Table 5). Mean differences for *PPD-D1* were greatest in all environments, with *PPD-D1a* genotypes heading an average of 2.0 to 5.6 days earlier than *PPD-D1b* genotypes. *PPD-A1a* genotypes were 0.8 to 3.9 days earlier than *PPD-A1b* genotypes across environments.

**Table 5:** Mean difference (days) between RIL having the AGS 2000 and 26R61 alleles at major flowering time loci.

Environments	*VRN-A1*	*VRN-B1*	PPD-A1	PPD-B1	PPD-D1
LA12	3.4***^a^	-7.0***	-3.9***	-1.4***	5.4***
GA12	3.4***	-3.7***	-3.1***	-2.0***	4.4***
NC12	3.0***	-2.4***	-3.8***	-2.9***	5.6***
NC13	1.5**	-1.1**	-2.3***	-1.7***	3.1***
NC14	1.3**	-0.5**	-0.8**	-0.8**	2.0***

Negative values indicate that the AGS 2000 alleles confer earlier flowering at field locations; Baton Rouge, LA, 2012; LA12, Plains, GA, 2012; GA12 Raleigh, NC, 2012; NC12, 2013; NC13, 2014; NC14.

^a^**, *** Level of significance at 0.01 and 0.001, respectively.

Examination of mean HD of multi-locus *PPD1* genotypes determined RIL having photoperiod sensitive alleles (SSS) in all three genomes headed significantly later than all other *PPD1* combinations across all environments (Table 6). The second latest flowering group were lines having only an insensitive allele at the *PPD-B1* locus (SIS). RILs possessing the *PPDA1a*, *PPD-B1b*, *PPD-D1b* (ISS) genotype were generally earlier than the *PPD-A1b*, *PPD-B1a*, *PPD-D1b* (SIS) group, although the difference was small and only significant in the LA12 environment. At the three NC environments, mean heading of RILs having only the *PPD-D1a* insensitive allele (SSI) was not significantly different from that of lines having multiple insensitive alleles. This was not the case in LA12 where lines with multiple alleles for insensitivity were earlier than lines possessing a single insensitive allele.

**Table 6:** Effect of *PPD1* gene combinations on heading dates in the field experiments.

*PPD-A1*, *PPD-B1*, and *PPD-D1* allele combination	Predicted phenotypes	Day of the year				
		LA12	GA12	NC12	NC13	NC14
A1a, B1a, D1a	I,I,I (21)	73.5	80.4	88.4	108.2	116
A1a, B1b, D1a	I,S,I (26)	72.2	79.6	87.6	107.2	115.4
A1b, B1a, D1a	S,I,I (27)	74.7	80.3	88.6	108	116.2
A1a, B1a, D1b	I,I,S (25)	75.8	81.9	89.4	108	116.3
A1b, B1b, D1a	S,S,I (24)	76.1	82	89.5	108.2	116.3
A1a, B1b, D1b	I,S,S (21)	77.6	82.4	91	109.3	118.3
A1b, B1a, D1b	S,I,S (16)	80.8	84.6	93.1	110.1	117.5
A1b, B1b, D1b	S,S,S (17)	88.8	92.8	105.2	118	120.1
	LSD^a^	2.5	2.0	2.3	1.8	1.3

Mean heading dates for AGS 2000 × 26R61 recombinant inbred lines having different *PPD1* gene combinations when grown at Baton Rouge, LA, 2012; LA12, Plains, GA, 2012; GA12 Raleigh, NC, 2012; NC12, 2013; NC13, 2014; NC14. Alleles with "a" designation confer day length insensitive phenotype (I) and "b" alleles are associated with day length sensitivity (S).

^a^ LSD = Least significant difference (*p*<0.05).

Similarly, mean HD for contrasting genotypes at the *VRN-A1* and *VRN-B1* loci were significantly different in all environments. The largest difference was observed for RIL having the *vrn-B1_del* allele that headed an average of 7.0 days earlier than *vrn-B1_non-del* genotypes at LA12. Significant mean HD differences for *vrn-B1_del* and *vrn-B1_non-del* were detected at the NC12, NC13 and NC14 environments although QTL at *VRN-B1* did not reach significance at these NC locations. Less variability was observed for the *VRN-A1* locus, with differences between *vrn-A1_2-copy* and *vrn-A1_3-copy* genotypes ranging from 1.3 to 3.4 days.

The interaction between *VRN-A1* and *VRN-B1* in the field was similar to that observed in the greenhouse. RILs having the *vrn-A1_3-copy* allele from AGS2000 and the intact *vrn-B1_non-del* allele from 26R61 headed significantly later at all locations than lines having either one or both of the weak winter alleles (Table 3). Mean HD of RILs having either the *vrn-A1_2-copy* or the *vrn-B1_del* allele or both did not differ in the three NC environments. However, RIL having the *vrn-B1*_*del* weak winter allele in combination with the *vrn-A1_3-copy* allele were earlier heading on average than RIL having an intact *vrn-B1_non-del* gene and *vrn-A1-2-copy* when grown in the LA12 environment. Mean HD of RIL having both weak winter alleles (*vrn-A1_2-copy* and *vrn-B1_del*) was significantly earlier than all other combinations at GA12 and LA12.

## Discussion

Winter wheat production extends north-south across most of the United States and breeding programs located in each state develop cultivars that are best suited to their specific set of target environments. In this study we investigated the genetic control of HD in a population derived from a cross between two winter wheat cultivars adapted to the southeastern United States. This production region is characterized by short periods of exposure to cold temperatures during winter that can be variable from year to year. In these environments, it is advantageous for plants to complete grain filling before the onset of high summer temperatures. Thus, selection for early maturity has been an important criteria for cultivar development. However, early flowering increases the risk of damage to sensitive floral structures and yield losses due to late spring freezes. Our analyses indicate that genetic variation at the major vernalization and photoperiod loci allows winter wheat breeders in these southern locations to time plant developmental stages to suit environmental conditions.

Winter wheat vernalization requirements can be variable requiring shorter or longer duration of cold exposure to induce flowering. Thus, critical flowering loci that are regulated by environmental factors are expected to interact variably across a range of environments that vary in intensity and duration of cold temperatures during winter. In our study, QTL by environment interaction was observed for the *VRN-A1* and *VRN-B1* loci. In the greenhouse experiment, the *VRN-A1* and *VRN-B1* loci did not have significant effect on HD in plants where vernalization requirement was satisfied by eight weeks of cold exposure. However, in the partial vernalization treatments (2W and 4W), QTL associated with *VRN-A1* and *VRN-B1* were each highly significant and together explained most of the variation in heading. Significant effects on HD were also observed when the populations were grown at field locations Baton Rouge, LA (Latitude: 30.54°), Plains, GA (Latitude: 32.05°) and Raleigh, NC (Latitude: 35.73°). Variation in winter and early spring temperatures were observed across years and locations (S2 Fig) and vernalization gene effects were greatest during the warmer winter and early spring of 2012. However, significant differences in HD were associated with the *VRN1* loci across all years and locations.

Vernalization requirement differences associated with the *VRN-A1* locus have been attributed to either mutations in coding sequences [18] or copy number differences [16]. The nonsynonymous mutation in exon 7 associated with early flowering in the cultivar Jagger [18] was not present in the 26R61 *vrn-A1_2-copy* allele in this study, as both parents carry the T nucleotide at this position. A study by Xiao et al. [35] determined that *TaGRP2*, an RNA binding protein, negatively regulates *VRN1* by directly binding to critical regions in the first intron. Recent characterization of the *VRN-D4* locus, which arose by duplication of *VRN-A1*, identified SNP in the conserved *TaGRP2* RIP-3 region that disrupts TaGRP2 binding to *VRN1* [32]. These same SNP were found in the common wheat cultivars Jagger and Claire, each of which has a single *VRN-A1* copy and reduced vernalization requirement. In the current study, a KASP assay designed to detect the SNP in the RIP-3 region did not reveal differences between the parents of our mapping population that both have the Triple Dirk C haplotype [32]. We did however, observe differences between AGS 2000 and 26R61 in exon 4 as well as gene copy number differences. Díaz et al. [16] determined that plants with increased *VRN-A1* copy number have an increased vernalization duration requirement such that longer periods of cold exposure were required to potentiate flowering. Similarly, we observed that the *vrn-A1_3-copy* allele was associated with later HD after partial vernalization in the greenhouse and at all field locations.

Our results demonstrate that the *vrn-B1_del* allele from AGS 2000 is associated with reduced vernalization requirement in lines having either two or three haploid copies of *VRN-A1*. The *vrn-B1_del* allele does not confer spring growth habit as lines having this variant are responsive to cold treatment. Four weeks of vernalization treatment accelerated heading in AGS 2000 and in the AP129E NIL having v*rn-B1_del* by 25 and 31 days, respectively, compared with non-vernalized plants. The mechanisms contributing to differences in vernalization requirement associated with the *VRN-B1* locus are not clear. The dominant *VRN1* genes have structural variations at critical promoter elements and large deletions in intron 1, which leads to loss of vernalization requirement [7,14]. Polymorphisms between AGS 2000 and 26R61 *vrn-B1* were not detected in regions thought to cause vernalization differences. Our comparative sequence analysis did not detect polymorphism between *vrn-B1* alleles at the TaGRP2 RIP-3 region or other known regulatory sites. In addition, polymorphism was not detected in exonic regions. AGS 2000 and 26R61 both had a single haploid *VRN-B1* copy, suggesting that flowering time variation was not due to gene dosage. The 36 bp deletion in intron 1 of the *vrn-B1_del* allele may play a role in reducing vernalization requirement. However, it is also possible that acceleration of flowering by short exposure to cold in this case is conferred by some yet to be described mechanism or is due to a linked locus. The Phytochrome C loci are located near the *VRN1* genes on group 5 chromosomes and have been shown to play a role in acceleration of flowering under long days [36].

The relative effects of *VRN-A1* and *VRN-B1* on HD in this study depended on the length of cold treatment. *VRN-A1* explained a larger percentage of variation in HD than *VRN-B1* and had a larger additive effect after 4W of cold treatment. In contrast, after two weeks of cold, the *VRN-B1* locus was most significant and had a larger additive effect (9.8 days) than *VRN-A1* (3.3 days). In addition, mean HD differences between the *VRN-B1* NIL pair were larger than that observed for the *VRN-A1* NIL pair in all partial vernalization treatments, particularly after the 2W treatment (33 days versus 20 days). These data suggest the 5B QTL region from AGS 2000 leads to more rapid transition to reproductive growth after short periods of exposure to cold temperatures than the *vrn-A1_2-copy* allele. Results of QTL analysis of the RIL population in the field are consistent with this observation. The difference in HD overserved at the LA12 environment for RIL having contrasting *VRN-B1* alleles (7 days) was twice the difference for RIL having contrasting *VRN-A1* alleles (3.4 days). However, at the NC locations, where plants were exposed to more cold days during winter, *VRN-B1* effects were smaller than that observed for *VRN-A1* (Table 5).

In this population, the number of RILs having two *vrn-A1* copies was almost twice that of lines having three *vrn-A1* copies. Selection again the *vrn-A1_3-copy* allele may have occurred during single seed descent of the mapping population. Plants were given four to six weeks of vernalization treatment in each generation of inbreeding, which was adequate to induce flowering in the parents and allowed for rapid population advancement. However, these shorter duration vernalization treatments would have resulted in delayed flowering of plants having the *vrn-B1_non-del* and *vrn-A1-3-copy* allele combination. Interestingly, significant segregation distortion of *vrn-B1* alleles was not observed. Thus, the cumulative effects of selection against very late flowering lines were greater for the VRN-A1 than the VRN-B1 locus.

In contrast with the QTL associated with the *VRN1* loci, direction and relative effects of the different *PPD1* insensitive genes were consistent across field environments although the magnitude of genetic effects varied. In all cases, the *PPD-D1a* insensitive allele had the greatest effect on early HD, followed by the *PPD-A1a*.*1* and the *PPD-B1a* alleles. The 26R61 parent of our population has the major *PPD-D1a* allele associated with a 2,089 bp deletion in the region upstream of the gene [12]. This *PPD-D1a* allele was reported to have the largest genetic effect on accelerating flowering time in current European wheat cultivars [37].

Comparison of the potency of *PPD1* genes in the literature can be complicated as recent molecular characterization has shown that multiple alleles are present at each locus [16,22,24,26]. The B-genome locus is particularly variable, with *PPD-B1a* alleles arising due to structural variation in the 5’ upstream region [24] as well as multiple gene duplication events leading to copy number variation [16,26]. Some studies showed that the *PPD-B1a* gene with a 308 bp insertion in the upstream region could be as strong as *PPD-D1a* under short photoperiod conditions [38,39]. The AGS 2000 parent of our population has the *PPD-B1a* allele associated with the Sonora/Timstein intercopy junction type and was estimated to have three gene copies. Díaz et al. [16] determined that an early flowering day neutral phenotype was associated with increased copy number of *PPD-B1*. In our study the three copy *PPD-B1* variant was associated with earlier heading date. However, the effect of *PPD-B1a* on HD in our population was consistently smaller than that observed for *PPD-D1a* and *PPD-A1a*. The *PPD-A1a* allele in AGS 2000 is characterized by a 1,085 bp deletion in the critical upstream region that was first reported in a Hokkaido cultivar ‘Chihokukomugi’ from Japan and designated as *PPD-A1a*.*1* [24]. Acceleration of heading due to the *PPD-A1a*.*1* allele in our population was intermediate to *PPD-D1a* and *PPD-B1a*. RIL having both *PPD-A1a* and *PPD-B1a* were generally earlier than RIL having either insensitive allele singly. However, RIL having multiple insensitive alleles were not significantly earlier than RIL possessing only *PPD-D1a*.

The widespread use of photoperiod insensitive alleles in winter wheat results in plants that are able to transition to flowering as soon as vernalization requirement is satisfied. In our experiment, this phenomenon was most apparent at the environments having the warmest winter/early spring temperatures. Although the experiment was conducted at the same NC location for three years, mean HD of the population was 18 and 26 days earlier at NC12 than NC13 and NC14, respectively. Not only were effects of vernalization loci larger in NC12, the genetic effects associated with the *PPD1* genes were also larger. The RIL population was not large enough to provide a good assessment of interactions among the five *VRN1* and *PPD1* genes segregating; however, some interesting interactions could be observed. At the LA12 and GA12 locations, 17 and 10 RIL, respectively, did not flower prior to the end of the experiments. Inspection of the multi-locus genotype data revealed that these extremely late RIL had the *vrn-A1_3-copy* and *vrn-B1_non-del* allele combination or had photoperiod sensitive alleles at all three *PPD1* loci in combination with one with one or both the *vrn-A1_3-copy* or *vrn-B1_non-del* alleles. Interestingly, photoperiod sensitive RIL having the both short vernalization alleles (*vrn-A1_2-copy* and *vrn-B1_non-del*) reached heading prior to the end of the experiments. These results implicate an important role of vernalization genes in control of plant development in these environments.

Allelic variation across two *VRN1* and three *PPD1* loci resulted in quantitative variation for heading date in our population. These data provide further evidence for the critical role of gene dosage and structural variation at *VRN1* and *PPD1* in timing of developmental stages in winter wheat in response to environmental cues. In order to define strategic breeding approaches to develop wheat cultivars adapted to growing regions experiencing warmer or more variable environments, it is important to assess the range of flowering time variation available to winter wheat breeders by characterizing the alleles of genes that modulate flowering time.

## Supporting Information

S1 Fig AGS 2000, 26R61, and Triple Dirk C *vrn-B1* Clustal sequence alignments. The 36 bp deletion spans from nucleotide base 5,400 to 5,436 based on Triple Dirk C (AY747604) *vrn-B1* reference sequence.
(TIF)

S2 Fig Growing degree days from January 1^st^ to April 30^th^ in Baton Rouge, LA, 2012; LA12, Plains, GA, 2012; GA12 Raleigh, NC, 2012; NC12, 2013; NC13, 2014; NC14. (TIF)

S3 Fig Position of major QTL for heading date. Linkage map of chromosomes harboring major QTL for heading date (hd) in AGS 2000 × 26R61 recombinant inbred lines grown in field experiments at Baton Rouge, LA, 2012; LA12, Plains, GA, 2012; GA12 Raleigh, NC, 2012; NC12, 2013; NC13, 2014; NC14.
(PDF)

S1 Table Primer pairs for amplifying the 13 kb *VRN-B1* gene by parts. Primer nucleotide positions are relative to the Triple Dirk C *VRN-B1* sequence (AY747604).
(PDF)

S2 Table *VRN-B1* primer walk sequencing primers. Primer nucleotide positions are relative to the Triple Dirk C *VRN-B1* sequence (AY747604).
(PDF)

S3 Table Linkage map constructed from the AGS 2000 by 26R61 mapping population including all DArT, SSR, STS and SNP markers. (PDF)

S4 Table Distribution of markers on linkage map of the AGS 2000 by 26R61 RIL mapping population. (PDF)

S5 Table Map location and genotypic data for parents and RILs for unique loci in the AGS 2000 by 26R61 mapping population. (XLSX)

S6 Table Heading date of AGS 2000, 26R61 and the RILs in the greenhouse and field. Days to heading were evaluated in the greenhouse following 8 weeks (8W), 4 weeks (4W) and 2 weeks (2W) of vernalization treatment. Heading date was recorded as day of year when lines were grown in the field in Baton Rouge, LA, Plains, GA and Raleigh, NC during 2012, 2013 and 2014.
(PDF)

## Abbreviations

HD: Heading Date
NIL: Near Isogenic Line
RIL: Recombinant Inbred Line
QTL: Quantitative Trait Loci
