# Supplementary figures and images for: Heading Date QTL in Winter Wheat (Triticum aestivum L.) Coincide with Major Developmental Genes VERNALIZATION1 and PHOTOPERIOD1
Source: PLoS One. 2016 May 10;11(5):e0154242. doi: 10.1371/journal.pone.0154242 (PMC4862677; doi:10.1371/journal.pone.0154242)

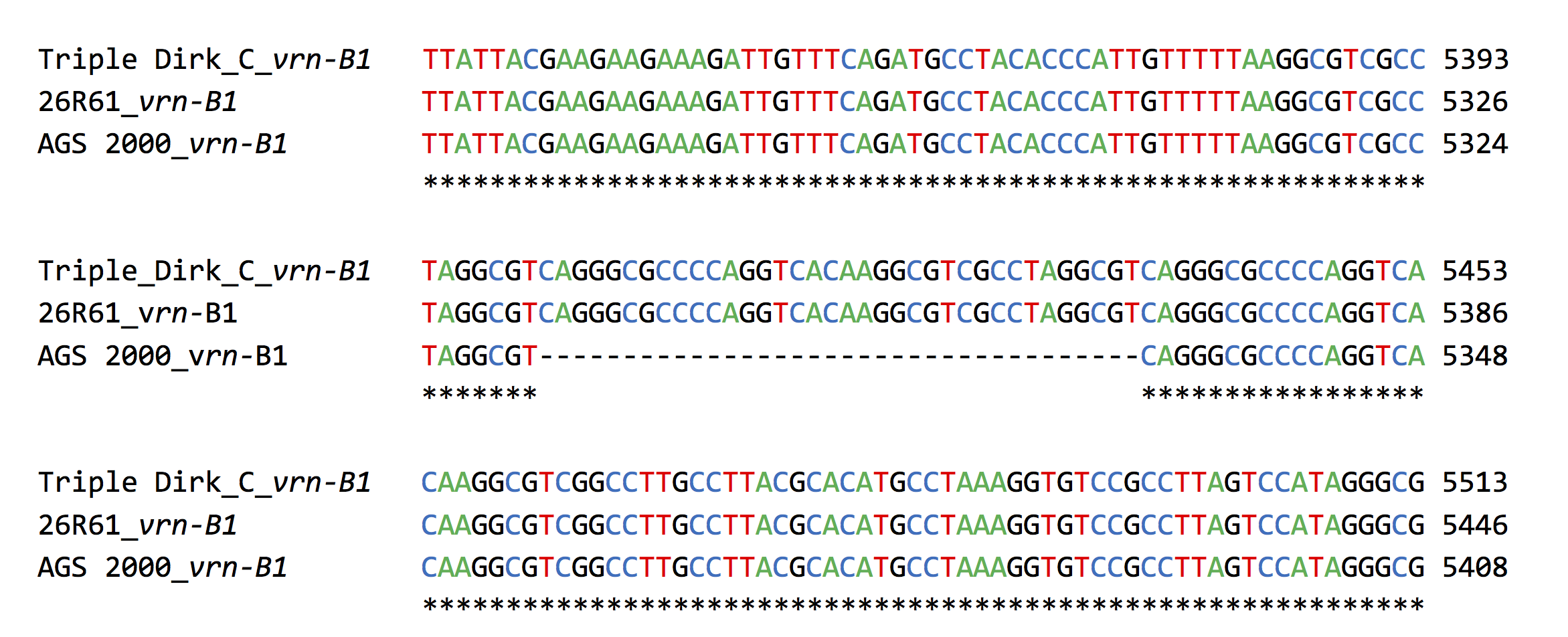

Supplement: S1 Fig — The 36 bp deletion spans from nucleotide base 5,400 to 5,436 based on Triple Dirk C (AY747604) vrn-B1 reference sequence. (TIF) [file pone.0154242.s001.tif]

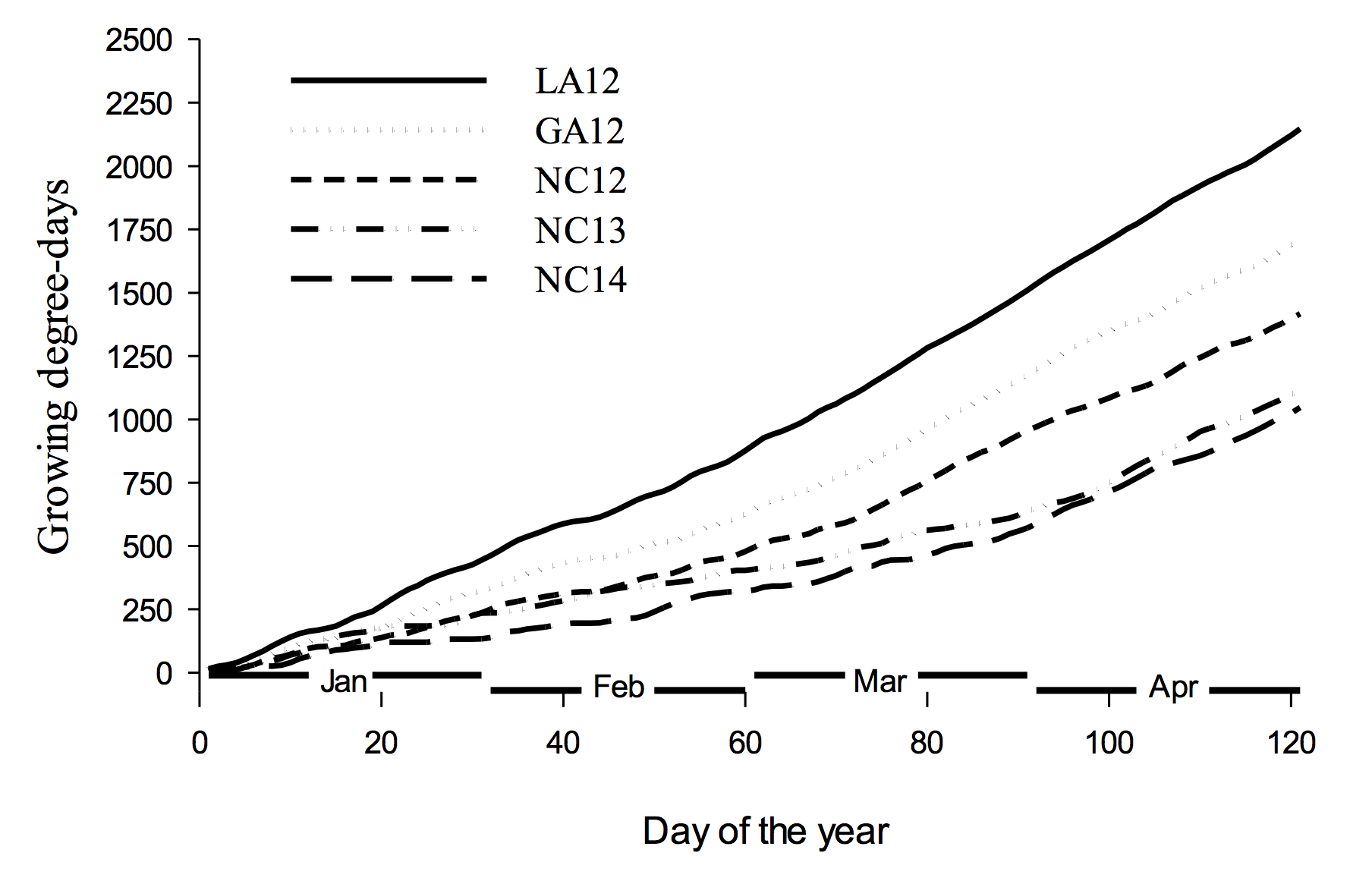

Supplement: S2 Fig — (TIF) [file pone.0154242.s002.tif]

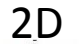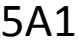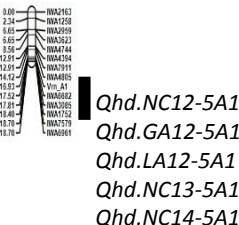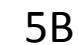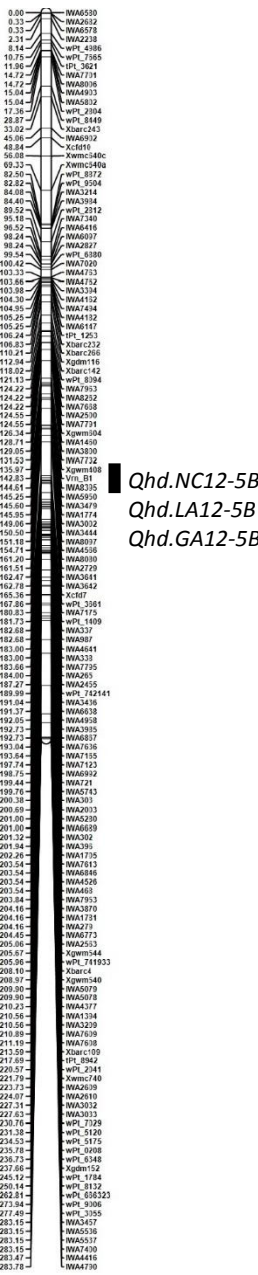

Supplement: S3 Fig — Linkage map of chromosomes harboring major QTL for heading date (hd) in AGS 2000 × 26R61 recombinant inbred lines grown in field experiments at Baton Rouge, LA, 2012; LA12, Plains, GA, 2012; GA12 Raleigh, NC, 2012; NC12, 2013; NC13, 2014; NC14. (PDF) [file pone.0154242.s003.pdf]
