# Supplementary material for: Heading Date QTL in Winter Wheat (Triticum aestivum L.) Coincide with Major Developmental Genes VERNALIZATION1 and PHOTOPERIOD1
Source: PLoS One. 2016 May 10;11(5):e0154242. doi: 10.1371/journal.pone.0154242 (PMC4862677; doi:10.1371/journal.pone.0154242)
Supplement: S1 Table — Primer nucleotide positions are relative to the Triple Dirk C VRN-B1 sequence (AY747604). (PDF) [file pone.0154242.s004.pdf]

S1 Table. Primer pairs for amplifying the 13 kb *VRN-B1* gene by parts. Primer nucleotide positions are relative to the Triple Dirk C *VRN-B1* sequence (AY747604).

Primer Name	Sequence 5'-3'	Expected Amplicon Size
vrnB1_1F	GCTCTTTCCCTTCTACTAGGC	0.83 kb
vrnB1_1R	CGAATCAACCAAACAGTGG	
vrnB1_2F	CTCTCCTCCCTCTCTTCCGC	6 kb
vrnB1_2R	TTGGTCACACATGTCATTCCTCT	
vrnB1_3F	ACGCACATGCCTAAAGGTGT	2.5 kb
vrnB1_3R	CGCGTCTCCCTGTGAGAAAT	
vrnB1_4F	TAGGCACGAGGAGGTGGTTA	6 kb
vrnB1_4R	GCAACCGCAACATACACCAG	
vrnB1_5F	GCATGTACATATGACGAGGA	1.79 kb
vrnB1_5R	ACATAAACAACACCCGCTC	
