# Supplementary material for: Heading Date QTL in Winter Wheat (Triticum aestivum L.) Coincide with Major Developmental Genes VERNALIZATION1 and PHOTOPERIOD1
Source: PLoS One. 2016 May 10;11(5):e0154242. doi: 10.1371/journal.pone.0154242 (PMC4862677; doi:10.1371/journal.pone.0154242)
Supplement: S2 Table — Primer nucleotide positions are relative to the Triple Dirk C VRN-B1 sequence (AY747604). (PDF) [file pone.0154242.s005.pdf]

S2 Table. *VRN-B1* primer walk sequencing primers. Primer nucleotide positions are relative to Triple Dirk C *VRN-B1* sequence (AY747604).

Sequencing Primer	Sequence 5'-3'	Nucleotide Position
vrn1_1	CGGATCGAGAACAAGATCAACC	531
vrn1_2	CCCAAGTGGAACGGTTAGGA	1051
vrn1_3	GGTTCTTGACAAAGTAAAGGAG	1551
vrn1_4	CTGTTTCTGCGCTGTTCATTTGG	2091
vrn1_5	CTGACATTGCAACCAACTTTACTC	2592
vrn1_6	GGCACGTACATGTAAGCAGATCC	3092
vrn1_7	AGCACAAGCAACAATGAAAGTG	3659
vrn1_8	CGGAGGGAGGTATCAGTGGT	4168
vrn1_9	TTTCATACCGAGATACGAAAGACC	4753
vrn1_10	AGACACGATGGATGGGAAATATCG	5255
vrn1_11	TTCTCCTCTCCAGGACAGTAGG	5755
vrn1_12	GCTCTACGACTCCTTTGATGATGC	6258
vrn1_13	CTCTTTACGATTGATGTGGTG	6758
vrn1_14	GTAGACCAAATTTCACATGGG	7258
vrn1_15	TTTAAGCTTCCTTCTCCCGC	7769
vrn1_16	CTCTCGCTTCAATCCTATGTTCG	8278
vrn1_17	TTTACGTAGGTGGTGGCAGTG	8803
vrn1_18	GCATCAACGTGGTGACTCTG	9058
vrn1_19	TTTCACATGGGCATTTATCTGC	9322
vrn1_20_R	CCGTCAGACGCTACCCTATA	9691
vrn1_21	GACAGGGAGAAGGTATAGGA	9822
vrn1_22	CTTTCTCTTATGCAGTATGGAC	10338
vrn1_23	GTGTGCATCAGTGACAATCAC	10897
vrn1_24	TTGAATATTTACCTCCACTGCAGC	11400
vrn1_25	AGCTCACTGAAACATATCAGATCC	11903
vrn1_26	TTTATTGACACAGATCCCTGTC	12423
