# Supplementary material for: Heading Date QTL in Winter Wheat (Triticum aestivum L.) Coincide with Major Developmental Genes VERNALIZATION1 and PHOTOPERIOD1
Source: PLoS One. 2016 May 10;11(5):e0154242. doi: 10.1371/journal.pone.0154242 (PMC4862677; doi:10.1371/journal.pone.0154242)
Supplement: S3 Table — (PDF) [file pone.0154242.s006.pdf]

S3 Table. Linkage map constructed from the AGS 2000 by 26R61 mapping population including all DArT, SSR, STS and SNP markers.

	Markers	Chromosome	Position cM
**Group 1**	IWA4241	1A	0
	IWA8622	1A	0.87
	Pm3_STS	1A	1.45
	Xgdm33	1A	2.32
	Xgwm33	1A	4.1
	IWA4240	1A	5.58
	IWA4505	1A	6.23
	IWA1481	1A	7.53
	IWA4033	1A	7.53
	IWA2452	1A	8.19
	IWA4644	1A	8.19
	Xwmc818	1A	9.44
	Xcfd15	1A	10.02
	Xgwm136	1A	12.73
	IWA3182	1A	16.03
	wPt_665724	1A	16.03
	wPt_6709	1A	16.61
	wPt_666537	1A	16.61
	wPt_730618	1A	16.61
	wPt_732520	1A	16.61
	wPt_734000	1A	16.61
	IWA6644	1A	16.92
	IWA5150	1A	17.25
	Xpsp2999	1A	17.89
	Xwmc329	1A	25.13
	wPt_743329	1A	28.18
	wPt_667558	1A	28.18
	wPt_669118	1A	28.18
	wPt_1862	1A	28.18
	IWA7050	1A	32.22
	IWA7377	1A	32.22
	IWA1387	1A	32.22
	IWA4163	1A	32.89
	IWA4164	1A	33.21
	IWA7796	1A	35.82
	wPt_731617	1A	42.29
	wPt_8172	1A	47.37
	IWA1142	1A	51.5
	IWA164	1A	51.5
	IWA5339	1A	51.5

IWA3399	1A	53.2
wPt_730356	1A	54.43
wPt_732546	1A	54.43
wPt_731806	1A	54.43
wPt_668214	1A	54.43
wPt_733007	1A	54.43
wPt_730148	1A	54.43
wPt_731807	1A	54.43
wPt_733779	1A	54.43
wPt_734216	1A	54.43
wPt_733464	1A	54.43
wPt_733858	1A	54.43
IWA3398	1A	55.7
IWA710	1A	55.7
IWA7421	1A	55.7
IWA2656	1A	55.7
IWA2922	1A	55.7
IWA2921	1A	55.7
IWA1450	1A	55.7
IWA3254	1A	55.7
IWA3347	1A	55.7
IWA3346	1A	55.7
IWA8307	1A	55.7
IWA3115	1A	55.7
IWA5080	1A	55.7
IWA1580	1A	55.7
IWA8615	1A	55.7
IWA2655	1A	55.7
wPt_668162	1A	56.65
wPt_669299	1A	56.65
wPt_668306	1A	56.65
wPt_666117	1A	56.65
wPt_7074	1A	56.65
wPt_667984	1A	59.73
wPt_666607	1A	59.73
wPt_6074	1A	59.73
wPt_3107	1A	60.96
wPt_733015	1A	61.26
Xgwm135	1A	63.99
IWA7173	1A	66.08
IWA5310	1A	66.71
IWA4291	1A	67.36
IWA4292	1A	67.36
IWA2995	1A	67.36
IWA5740	1A	67.36
IWA4179	1A	67.67
IWA7577	1A	68.84

IWA3134	1A	69.13
IWA1609	1A	69.13
IWA1593	1A	69.13
IWA3934	1A	69.13
IWA3613	1A	69.13
IWA7898	1A	69.13
IWA6709	1A	69.13
IWA3477	1A	69.13
IWA1594	1A	69.44
IWA3821	1A	69.76
IWA3822	1A	69.76
IWA5777	1A	69.76
IWA6707	1A	71.07
IWA5174	1A	71.38
IWA6708	1A	71.38
IWA4283	1A	71.69
IWA163	1A	72.29
IWA6553	1A	72.29
IWA162	1A	72.29
IWA7869	1A	72.29
IWA7871	1A	72.29
IWA7868	1A	72.29
IWA7573	1A	72.29
IWA5169	1A	72.29
IWA3419	1A	72.29
IWA8101	1A	72.29
Xcfa2129	1A	77.87
IWA4955	1A	86.41
IWA6341	1A	86.41
IWA339	1A	88.5
IWA7145	1A	88.5
IWA3614	1A	88.5
IWA3859	1A	88.5
IWA5493	1A	88.5
IWA3405	1A	88.84
IWA531	1A	90.61
IWA530	1A	90.95
IWA4538	1A	94.19
IWA1081	1A	94.89
IWA577	1A	95.88
IWA578	1A	95.88
IWA3434	1A	97.48
IWA3435	1A	97.48
IWA5047	1A	98.76
IWA5046	1A	99.1
IWA136	1A	99.41
IWA135	1A	99.41

IWA691	1A	100.04
IWA1195	1A	100.04
IWA1790	1A	100.04
IWA8334	1A	100.68
IWA3962	1A	100.68
IWA3060	1A	100.68
IWA3195	1A	101
Xcfa2219	1A	103.55
IWA1119	1A	105.09
IWA1403	1A	105.09
IWA1587	1A	105.09
IWA3485	1A	105.09
IWA5910	1A	105.09
IWA2818	1A	105.09
IWA4713	1A	105.09
IWA3783	1A	105.09
IWA7315	1A	105.09
IWA735	1A	105.39
IWA4931	1A	105.39
IWA2784	1A	105.39
IWA2783	1A	105.39
IWA1837	1A	105.39
IWA2404	1A	105.39
IWA2405	1A	105.39
IWA7591	1A	105.39
IWA7924	1A	105.39
IWA3486	1A	105.39
IWA8135	1A	105.39
IWA1015	1A	105.39
IWA1619	1A	105.39
IWA1118	1A	105.39
IWA7428	1A	105.39
IWA6253	1A	105.39
IWA7316	1A	105.39
IWA7707	1A	105.39
IWA886	1A	105.39
IWA6975	1A	106.02
IWA5822	1A	106.02
IWA6081	1A	106.02
IWA1225	1A	106.02
Xcfa2147	1A	107.67
Xgwm99	1A	107.96
wPt_7339	1A	108.84
tPt_1012	1A	108.84
wPt_0128	1A	108.84
wPt_666616	1A	108.84
wPt_2847	1A	108.84

wPt_9938	1A	108.84
wPt_5660	1A	108.84
wPt_733904	1A	108.84
wPt_730885	1A	108.84
wPt_733811	1A	108.84
wPt_667252	1A	108.84
wPt_669499	1A	110.77
Xgwm497	1A	111.72
Xwmc716	1A	111.72
wPt_4897	1A	112.3
wPt_5316	1A	112.3
wPt_5367	1A	112.91
IWA6152	1A	115.26
Xwmc59	1A	116.87
IWA2994	1A	121.79
IWA5491	1A	122.11
IWA3378	1A	124.42
wPt_734285	1A	133.88
wPt_1010	1A	133.88
tPt_1419	1A	133.88
wPt_5077	1A	133.88
wPt_0497	1A	133.88
wPt_733820	1A	133.88
wPt_666087	1A	133.88
wPt_6754	1A	133.88
wPt_732881	1A	133.88
wPt_8644	1A	133.88
wPt_734288	1A	133.88
wPt_732377	1A	133.88
wPt_0164	1A	133.88
wPt_730902	1A	133.88
wPt_6005	1A	136.69
wPt_668205	1A	139.55
IWA8523	1A	140.74
IWA7290	1A	140.74
IWA4518	1A	140.74
IWA5407	1A	141.05
IWA2327	1A	141.05
IWA4271	1A	141.05
IWA1306	1A	141.67
IWA1644	1A	144.71
IWA6916	1A	144.71
IWA4123	1A	146.33
IWA4122	1A	146.33
IWA4120	1A	146.33
IWA3661	1A	146.33
IWA5405	1A	146.33

	Xbarc17	1A	147.58
**Group 2**	IWA1399	1B	0
	IWA6062	1B	0
	IWA8148	1B	0
	IWA5348	1B	1.78
	IWA6063	1B	1.78
	IWA8081	1B	1.78
	IWA2221	1B	2.06
	IWA7119	1B	2.06
	IWA8082	1B	2.06
	wPt_1573	1B	2.95
	wPt_1248	1B	4.17
	IWA3945	1B	4.78
	IWA6107	1B	4.78
	IWA1953	1B	4.78
	IWA491	1B	5.08
	IWA4203	1B	5.37
	IWA4204	1B	5.37
	IWA6917	1B	5.37
	IWA7560	1B	5.37
	IWA7982	1B	5.37
	Xwmc419	1B	5.68
	wPt_5485	1B	7.51
	IWA515	1B	8.11
	IWA854	1B	8.42
	IWA540	1B	9.06
	IWA3713	1B	9.66
	IWA7275	1B	9.66
	IWA6945	1B	9.66
	IWA4089	1B	9.96
	IWA6731	1B	9.96
	IWA3712	1B	9.96
	IWA6133	1B	9.96
	IWA6926	1B	9.96
	IWA1889	1B	10.58
	IWA1890	1B	10.58
	IWA2920	1B	11.51
	IWA4999	1B	11.51
	IWA4361	1B	11.51
	IWA4007	1B	11.51
	IWA8111	1B	11.51
	IWA5228	1B	11.51
	IWA5229	1B	11.51
	IWA6688	1B	11.51
	IWA5227	1B	11.51
	IWA3684	1B	11.51
	IWA2717	1B	11.51

IWA2626	1B	12.15
IWA4819	1B	12.15
IWA2315	1B	12.47
IWA6558	1B	12.47
wPt_8279	1B	13.11
wPt_8280	1B	14.36
wPt_3227	1B	16.29
wPt_0506	1B	16.9
wPt_0202	1B	16.9
IWA7214	1B	17.49
IWA3076	1B	17.49
IWA3189	1B	19.47
IWA5382	1B	20.13
IWA5383	1B	20.13
IWA3120	1B	20.78
IWA3384	1B	20.78
IWA4488	1B	20.78
IWA8313	1B	21.75
IWA1164	1B	23
IWA579	1B	23.3
wPt_9857	1B	24.19
rPt_7906	1B	25.42
wPt_729805	1B	31.16
IWA3017	1B	32.92
IWA367	1B	33.58
IWA7178	1B	33.58
IWA7179	1B	33.58
IWA8379	1B	33.58
IWA4875	1B	33.58
IWA8398	1B	33.58
IWA7037	1B	33.58
IWA858	1B	33.58
IWA2411	1B	33.58
IWA255	1B	33.58
IWA5769	1B	33.58
IWA2308	1B	33.87
IWA3341	1B	34.17
IWA368	1B	34.17
IWA5186	1B	34.17
IWA5861	1B	34.48
IWA5862	1B	34.48
IWA8246	1B	34.48
IWA5749	1B	35.13
IWA5915	1B	35.46
wPt_9032	1B	37.37
wPt_7066	1B	38.27
wPt_6975	1B	39.49

	wPt_1403	1B	40.41
	IWA5352	1B	42.81
	IWA8507	1B	43.13
	IWA3097	1B	45.33
	IWA7422	1B	46.38
	wPt_9809	1B	48.77
	IWA695	1B	50.05
	wPt_2205	1B	50.68
	wPt_5907	1B	50.68
	wPt_729773	1B	53.28
	IWA8542	1B	57.58
	wPt_4688	1B	59.93
	IWA1092	1B	64.19
	IWA7992	1B	71.66
	IWA8332	1B	71.66
	IWA4525	1B	88.98
	IWA6512	1B	92.84
	IWA2077	1B	92.84
	IWA2279	1B	92.84
	IWA2928	1B	92.84
	IWA6647	1B	92.84
	IWA2078	1B	93.16
**Group 3**	wPt_744571	1D1	0
	wPt_3636	1D1	24.26
	wPt_4711	1D1	25.51
	wPt_730879	1D1	25.51
	wPt_732144	1D1	26.13
	wPt_7491	1D1	27.4
	wPt_730894	1D1	28.65
	Xbarc62	1D1	29.88
	wPt_669681	1D1	29.88
	wPt_667395	1D1	29.88
	wPt_4988	1D1	29.88
	wPt_7697	1D1	29.88
	wPt_740692	1D1	29.88
	wPt_729788	1D1	29.88
	wPt_4427	1D1	29.88
	wPt_666963	1D1	29.88
	wPt_6560	1D1	29.88
	wPt_730172	1D1	29.88
	wPt_730605	1D1	29.88
	wPt_3945	1D1	29.88
	wPt_731020	1D1	29.88
	wPt_7957	1D1	29.88
	wPt_732575	1D1	29.88
	wPt_730475	1D1	29.88
	wPt_665749	1D1	29.88

	wPt_668079	1D1	29.88
	wPt_730718	1D1	29.88
	wPt_665360	1D1	29.88
	wPt_8545	1D1	29.88
	wPt_665327	1D1	29.88
	wPt_734132	1D1	29.88
	wPt_671947	1D1	29.88
	wPt_7057	1D1	29.88
	wPt_2839	1D1	30.78
	wPt_7215	1D1	32.04
	wPt_7711	1D1	33.59
	wPt_1263	1D1	33.59
	wPt_6503	1D1	33.59
	wPt_7092	1D1	33.59
	wPt_7421	1D1	33.59
	wPt_2312	1D1	33.59
	wPt_1531	1D1	33.59
	wPt_8321	1D1	33.59
	Xwmc728b	1D1	34.18
	wPt_5721	1D1	45.21
	wPt_5915	1D1	45.54
	wPt_8866	1D1	45.54
	wPt_2968	1D1	45.54
	wPt_4497	1D1	46.94
	wPt_667092	1D1	49.56
	wPt_5253	1D1	51.96
	wPt_9028	1D1	53.21
	wPt_734314	1D1	53.21
	wPt_3950	1D1	53.21
	Xwmc728a	1D1	59.5
**Group 4**	wPt_734229	1D2	0
	IWA165	1D2	16.19
	IWA1193	1D2	16.5
	IWA1192	1D2	16.5
	wPt_4671	1D2	16.8
	wPt_668040	1D2	16.8
	wPt_666832	1D2	16.8
	wPt_664609	1D2	16.8
	wPt_671545	1D2	16.8
	wPt_9380	1D2	16.8
	wPt_0413	1D2	16.8
	Xbarc229	1D2	18.29
	Xcfd59	1D2	22.73
	wPt_6316	1D2	29.06
	wPt_8854	1D2	38.95
	IWA7702	1D2	51.83
	IWA3549	1D2	52.16

	IWA3547	1D2	52.49
	IWA8156	1D2	53.82
	IWA5514	1D2	54.11
	IWA6805	1D2	54.11
	IWA8173	1D2	54.11
	IWA7675	1D2	54.43
	IWA2132	1D2	54.43
	IWA3242	1D2	54.77
	wPt_671869	1D2	56.1
	Xcfd27	1D2	58.63
**Group 5**	Xbarc212	2A	0
	Xbarc124	2A	2.4
	wPt_4533	2A	5.76
	Xgwm636	2A	8.17
	Xwmc667	2A	9.65
	wPt_4197	2A	10.23
	wPt_5647	2A	10.51
	tPt_1041	2A	10.51
	IWA7736	2A	11.14
	IWA4989	2A	11.79
	IWA6922	2A	11.79
	IWA2426	2A	12.41
	IWA6391	2A	12.41
	IWA5340	2A	12.41
	IWA5341	2A	12.41
	IWA1511	2A	12.41
	IWA2425	2A	12.41
	IWA2427	2A	12.41
	IWA5424	2A	12.41
	IWA1562	2A	12.41
	IWA6745	2A	12.7
	IWA5423	2A	12.7
	IWA1512	2A	12.7
	IWA2428	2A	12.7
	IWA5342	2A	12.7
	IWA1563	2A	12.7
	Xgwm359	2A	31.44
	IWA4441	2A	36.2
	IWA3235	2A	36.89
	IWA2059	2A	37.88
	IWA7410	2A	37.88
	IWA2696	2A	38.23
	tPt_9405	2A	38.95
	wPt_8490	2A	38.95
	wPt_740658	2A	38.95
	Xwmc598	2A	46.73
	wPt_8068	2A	54.91

wPt_6711	2A	56.12
wPt_0568	2A	56.12
Ppd_A1	2A	62.22
IWA4216	2A	68.26
IWA4217	2A	68.26
IWA4212	2A	68.58
IWA4213	2A	68.58
IWA4214	2A	68.58
IWA4215	2A	68.58
wPt_2384	2A	72.47
wPt_6139	2A	73.76
IWA6250	2A	80.68
IWA6566	2A	86.92
IWA2730	2A	86.92
IWA8424	2A	86.92
IWA2067	2A	86.92
IWA2731	2A	86.92
IWA6564	2A	86.92
IWA6565	2A	86.92
Xwmc522	2A	87.23
wPt_9320	2A	88.43
IWA8491	2A	93.11
IWA3802	2A	93.11
IWA314	2A	93.11
IWA3803	2A	93.11
IWA3569	2A	93.11
IWA4027	2A	95.85
IWA2245	2A	97.86
IWA1256	2A	97.86
IWA2531	2A	97.86
IWA4026	2A	97.86
IWA5378	2A	99.18
Xgwm95	2A	99.82
IWA3842	2A	101.03
IWA6753	2A	101.65
IWA3839	2A	101.65
Xbarc15	2A	103.49
IWA5068	2A	105.72
IWA812	2A	106.03
IWA32	2A	106.03
IWA33	2A	106.03
IWA69	2A	106.03
IWA2807	2A	106.31
IWA70	2A	106.31
IWA71	2A	106.31
IWA3151	2A	106.31
IWA7947	2A	108.09

IWA3417	2A	108.09
wPt_2473	2A	112.81
tPt_8937	2A	115.41
wPt_3114	2A	115.41
IWA2612	2A	120.75
Xgwm47	2A	135.96
Xgwm312	2A	146.53
IWA7433	2A	146.82
IWA7148	2A	146.82
IWA2884	2A	146.82
IWA5214	2A	146.82
IWA5215	2A	146.82
IWA5463	2A	146.82
IWA6845	2A	146.82
IWA7540	2A	146.82
IWA6499	2A	146.82
IWA6503	2A	146.82
wPt_3244	2A	146.82
IWA5216	2A	146.82
IWA6844	2A	146.82
IWA6286	2A	146.82
IWA5733	2A	146.82
IWA7593	2A	148.38
IWA4375	2A	148.69
IWA7864	2A	148.69
IWA4373	2A	148.69
IWA2157	2A	148.69
IWA3629	2A	149
IWA173	2A	149.96
wPt_5865	2A	154.06
IWA6155	2A	163.15
IWA5066	2A	164.38
IWA6931	2A	164.38
IWA1539	2A	164.38
IWA5959	2A	165.36
IWA2938	2A	165.68
IWA5685	2A	169.66
IWA5686	2A	169.98
IWA4336	2A	170.3
IWA5872	2A	170.64
IWA7412	2A	186.2
wPt_0118	2A	189.54
wPt_0801	2A	192.04
wPt_9793	2A	194.71
IWA1348	2A	195.67
IWA318	2A	196.63
IWA6839	2A	196.63

	IWA319	2A	196.94
	IWA5759	2A	202.85
	IWA3809	2A	204.48
	IWA2983	2A	204.48
	IWA5082	2A	204.48
	IWA551	2A	204.48
	IWA5894	2A	204.48
	IWA4461	2A	205.79
	IWA2777	2A	206.13
	IWA3688	2A	206.13
	wPt_6662	2A	208.95
	wPt_665972	2A	210.19
	Xgwm311	2A	214.75
	Xgwm382b	2A	215.32
	wPt_2858	2A	215.32
	wPt_1615	2A	215.32
	wPt_9586	2A	215.61
	IWA5364	2A	215.61
	wPt_668261	2A	219.52
	wPt_730757	2A	220.17
	wPt_800788	2A	223.97
	wPt_664140	2A	227.28
	wPt_7739	2A	229.6
	wPt_741201	2A	229.9
	IWA2778	2A	229.9
	wPt_799683	2A	229.9
	wPt_741584	2A	229.9
	wPt_669355	2A	229.9
	IWA4491	2A	230.84
	IWA4493	2A	230.84
	IWA4192	2A	231.16
	IWA6963	2A	231.16
	IWA7327	2A	231.16
	IWA5879	2A	231.16
	IWA4458	2A	231.16
	IWA4459	2A	231.16
	IWA7885	2A	231.16
	Xgwm265	2A	246.33
**Group 6**	IWA6481	2B	0
	wPt_733077	2B	20.47
	wPt_731381	2B	20.47
	wPt_733314	2B	20.79
	IWA7545	2B	33.25
	IWA6219	2B	33.57
	Xwmc264	2B	34.2
	wPt_2106	2B	35.12
	wPt_3565	2B	38.7

wPt_6158	2B	38.7
wPt_9274	2B	41.91
wPt_9230	2B	42.83
wPt_7672	2B	42.83
wPt_0100	2B	43.42
IWA4953	2B	44.05
wPt_3390	2B	45.06
wPt_4916	2B	46.02
wPt_744022	2B	46.02
Xwmc382	2B	46.61
IWA6262	2B	47.19
IWA6263	2B	47.19
IWA6008	2B	53.6
IWA1799	2B	55.73
wPt_7995	2B	56.76
IWA5772	2B	58.18
Xwmc243	2B	72.88
Xwmc154	2B	77.93
IWA2117	2B	81.88
IWA1929	2B	81.88
IWA5708	2B	81.88
IWA1360	2B	82.57
IWA6474	2B	82.9
wPt_3983	2B	83.22
wPt_4997	2B	83.22
wPt_3561	2B	83.53
wPt_9423	2B	83.83
wPt_8072	2B	83.83
wPt_743630	2B	83.83
wPt_1489	2B	83.83
wPt_9402	2B	83.83
wPt_7619	2B	83.83
wPt_9668	2B	83.83
wPt_7985	2B	83.83
wPt_4664	2B	84.12
IWA6048	2B	84.73
IWA6085	2B	84.73
IWA1930	2B	84.73
IWA4285	2B	84.73
IWA889	2B	85.03
Xbarc200	2B	86.81
IWA1359	2B	88.39
Ppd_B1	2B	94.63
IWA4554	2B	99.22
IWA4652	2B	99.22
IWA4420	2B	99.22
IWA6026	2B	99.22

IWA6739	2B	99.22
IWA7069	2B	99.22
IWA6452	2B	101.08
IWA6893	2B	101.69
IWA5697	2B	101.69
IWA4421	2B	101.69
IWA6740	2B	101.69
IWA6943	2B	101.69
wPt_2600	2B	102.62
Xwmc770	2B	104.41
wPt_666389	2B	105.29
wPt_8492	2B	106.24
wPt_1064	2B	106.55
wPt_5556	2B	107.15
wPt_6192	2B	107.15
wPt_4125	2B	107.15
wPt_7757	2B	107.15
wPt_5672	2B	107.15
IWA8083	2B	107.45
IWA1087	2B	109.05
tPt_0043	2B	111.08
wPt_6174	2B	112.09
tPt_9065	2B	112.75
IWA8182	2B	113.35
Xgwm148	2B	113.35
IWA608	2B	113.64
IWA8381	2B	113.64
IWA5392	2B	113.64
IWA6069	2B	113.64
IWA6364	2B	113.64
IWA2312	2B	113.64
IWA2887	2B	113.64
IWA4456	2B	113.64
IWA2556	2B	113.64
IWA2557	2B	113.64
IWA4655	2B	113.64
IWA5560	2B	113.64
IWA1665	2B	113.64
IWA4531	2B	113.64
IWA7029	2B	113.64
IWA3081	2B	113.64
IWA5916	2B	113.64
IWA4135	2B	113.64
IWA607	2B	113.93
IWA762	2B	113.93
IWA3564	2B	113.93
IWA295	2B	113.93

IWA763	2B	113.93
IWA4673	2B	113.93
IWA5753	2B	113.93
IWA5818	2B	113.93
IWA7567	2B	113.93
IWA1664	2B	113.93
IWA3824	2B	113.93
IWA3329	2B	113.93
IWA1114	2B	113.93
IWA4532	2B	113.93
IWA3080	2B	113.93
IWA8367	2B	113.93
wPt_6477	2B	113.93
Xbarc183b	2B	114.22
BE444297	2B	115.39
IWA6509	2B	115.69
IWA7076	2B	115.69
IWA7661	2B	115.69
IWA3126	2B	115.69
IWA5147	2B	115.69
IWA1204	2B	115.69
IWA2671	2B	115.69
IWA328	2B	115.69
IWA5377	2B	115.69
IWA2670	2B	115.97
IWA429	2B	115.97
IWA1763	2B	115.97
IWA2624	2B	115.97
IWA6838	2B	115.97
IWA8221	2B	115.97
IWA897	2B	115.97
IWA3127	2B	115.97
IWA6427	2B	115.97
IWA50	2B	116.28
IWA2673	2B	116.28
IWA6547	2B	116.28
IWA7951	2B	117.15
IWA3621	2B	117.15
IWA4984	2B	117.15
IWA5149	2B	117.15
IWA6664	2B	117.15
IWA3213	2B	117.15
IWA5059	2B	117.15
IWA1179	2B	117.15
IWA1059	2B	117.15
IWA1938	2B	117.15
IWA3657	2B	117.15

IWA6819	2B	117.15
IWA4102	2B	117.46
IWA3428	2B	117.46
IWA5038	2B	117.46
IWA5811	2B	117.46
IWA6075	2B	117.46
IWA5464	2B	117.46
IWA7263	2B	117.76
IWA6135	2B	117.76
IWA6462	2B	117.76
IWA6136	2B	117.76
IWA6476	2B	118.05
IWA6830	2B	118.05
IWA2977	2B	118.05
IWA4983	2B	118.05
IWA6875	2B	118.05
IWA6818	2B	118.05
IWA3622	2B	118.05
IWA3656	2B	118.05
IWA776	2B	118.05
IWA1237	2B	118.05
IWA439	2B	118.05
IWA6781	2B	118.05
IWA3258	2B	118.63
IWA7269	2B	118.63
IWA8517	2B	118.63
IWA5436	2B	118.63
IWA6209	2B	118.63
IWA7520	2B	118.63
IWA2714	2B	118.63
IWA3861	2B	118.63
IWA6918	2B	118.63
IWA5723	2B	118.63
IWA2939	2B	118.63
IWA3210	2B	118.63
IWA207	2B	118.63
IWA4752	2B	118.63
IWA6437	2B	118.63
IWA8244	2B	118.92
IWA535	2B	118.92
IWA2766	2B	118.92
IWA4100	2B	118.92
IWA1980	2B	118.92
IWA1981	2B	118.92
IWA3860	2B	118.92
IWA3862	2B	118.92
IWA3817	2B	118.92

IWA7251	2B	118.92
IWA2760	2B	118.92
IWA2940	2B	118.92
IWA3828	2B	118.92
IWA4751	2B	118.92
IWA7252	2B	118.92
IWA7253	2B	118.92
IWA7822	2B	118.92
IWA8112	2B	119.21
IWA2025	2B	119.21
IWA5017	2B	119.21
IWA2024	2B	119.21
IWA4134	2B	119.21
IWA5261	2B	119.21
IWA6009	2B	119.21
IWA433	2B	119.21
IWA4696	2B	119.21
IWA3942	2B	119.21
IWA7499	2B	119.21
IWA6438	2B	119.21
IWA4473	2B	119.21
IWA6639	2B	119.21
IWA5658	2B	119.21
IWA5654	2B	119.21
IWA6216	2B	119.21
IWA7489	2B	119.21
IWA3696	2B	119.21
IWA5678	2B	119.21
IWA5926	2B	119.21
IWA3452	2B	119.21
IWA1935	2B	119.21
IWA4399	2B	119.21
IWA3153	2B	119.21
IWA310	2B	119.21
IWA5575	2B	119.21
IWA3236	2B	119.21
IWA772	2B	119.21
IWA838	2B	119.21
IWA1215	2B	119.21
IWA1217	2B	119.21
IWA2030	2B	119.21
IWA2183	2B	119.21
IWA2236	2B	119.21
IWA2464	2B	119.21
IWA2465	2B	119.21
IWA2530	2B	119.21
IWA2544	2B	119.21

IWA2972	2B	119.21
IWA3045	2B	119.21
IWA3618	2B	119.21
IWA3889	2B	119.21
IWA4140	2B	119.21
IWA4189	2B	119.21
IWA4474	2B	119.21
IWA4660	2B	119.21
IWA4879	2B	119.21
IWA4882	2B	119.21
IWA4965	2B	119.21
IWA4982	2B	119.21
IWA5117	2B	119.21
IWA5168	2B	119.21
IWA5256	2B	119.21
IWA5260	2B	119.21
IWA5262	2B	119.21
IWA5290	2B	119.21
IWA5600	2B	119.21
IWA5610	2B	119.21
IWA5611	2B	119.21
IWA5927	2B	119.21
IWA5961	2B	119.21
IWA6000	2B	119.21
IWA6240	2B	119.21
IWA6539	2B	119.21
IWA6607	2B	119.21
IWA6921	2B	119.21
IWA6929	2B	119.21
IWA6948	2B	119.21
IWA7103	2B	119.21
IWA7146	2B	119.21
IWA7238	2B	119.21
IWA7379	2B	119.21
IWA7684	2B	119.21
IWA8454	2B	119.49
IWA837	2B	119.49
IWA31	2B	119.49
IWA326	2B	119.49
Xcfa2278	2B	119.49
IWA7420	2B	119.49
IWA2543	2B	119.49
IWA3453	2B	119.49
IWA1661	2B	119.49
IWA2050	2B	119.49
IWA3554	2B	119.49
IWA3948	2B	119.49

IWA5247	2B	119.49
IWA5248	2B	119.49
IWA6970	2B	119.49
IWA2625	2B	119.49
IWA4014	2B	119.49
IWA7919	2B	119.49
IWA2349	2B	119.49
IWA5653	2B	119.49
IWA4136	2B	119.49
IWA4224	2B	119.49
IWA4881	2B	119.49
IWA8227	2B	119.49
IWA1309	2B	119.49
IWA6016	2B	119.49
IWA1550	2B	119.49
IWA671	2B	119.49
IWA2081	2B	119.49
IWA7215	2B	119.49
IWA6966	2B	119.49
IWA4880	2B	119.49
IWA742	2B	119.49
IWA2739	2B	119.49
IWA4464	2B	119.49
IWA586	2B	119.49
IWA587	2B	119.49
IWA5131	2B	119.49
IWA5259	2B	119.49
IWA5263	2B	119.49
IWA1392	2B	119.49
IWA6505	2B	119.49
IWA6723	2B	119.49
IWA7959	2B	119.49
IWA7204	2B	119.49
IWA697	2B	119.49
IWA1056	2B	119.49
IWA1216	2B	119.49
IWA1549	2B	119.49
IWA2665	2B	119.49
IWA2899	2B	119.49
IWA3626	2B	119.49
IWA3648	2B	119.49
IWA3734	2B	119.49
IWA3840	2B	119.49
IWA3858	2B	119.49
IWA3865	2B	119.49
IWA3995	2B	119.49
IWA4106	2B	119.49

IWA4107	2B	119.49
IWA4128	2B	119.49
IWA4517	2B	119.49
IWA4541	2B	119.49
IWA4659	2B	119.49
IWA4822	2B	119.49
IWA5077	2B	119.49
IWA5090	2B	119.49
IWA5091	2B	119.49
IWA5659	2B	119.49
IWA5741	2B	119.49
IWA5794	2B	119.49
IWA5983	2B	119.49
IWA6003	2B	119.49
IWA6215	2B	119.49
IWA6265	2B	119.49
IWA6769	2B	119.49
IWA7015	2B	119.49
IWA7195	2B	119.49
IWA7312	2B	119.49
IWA7376	2B	119.49
IWA7524	2B	119.49
IWA8368	2B	119.49
Xbarc230	2B	119.79
wPt_6242	2B	119.79
IWA169	2B	120.07
IWA170	2B	120.36
IWA7821	2B	120.36
IWA829	2B	120.36
IWA771	2B	120.36
IWA777	2B	120.36
IWA869	2B	120.36
IWA874	2B	120.36
IWA1102	2B	120.36
IWA1127	2B	120.36
IWA1128	2B	120.36
IWA1129	2B	120.36
IWA1130	2B	120.36
IWA1131	2B	120.36
IWA1177	2B	120.36
IWA1188	2B	120.36
IWA1229	2B	120.36
IWA4605	2B	120.36
wPt_0079	2B	123.31
wPt_665920	2B	136.18
wPt_7064	2B	151.66
wPt_8390	2B	151.95

wPt_666459	2B	166.27
wPt_2430	2B	176.4
wPt_4889	2B	178.23
wPt_5878	2B	178.23
wPt_4199	2B	178.53
IWA6317	2B	179.42
IWA77	2B	179.42
IWA1393	2B	179.42
IWA2294	2B	179.42
IWA4275	2B	180.02
IWA2950	2B	180.63
IWA6453	2B	180.63
IWA3973	2B	180.63
IWA6076	2B	180.63
IWA2924	2B	180.63
IWA5789	2B	180.63
IWA5415	2B	180.63
IWA2131	2B	180.63
IWA4948	2B	180.95
IWA4356	2B	180.95
IWA242	2B	181.91
IWA8261	2B	182.21
IWA5141	2B	182.21
IWA243	2B	182.51
IWA2512	2B	183.14
IWA5008	2B	183.77
IWA5128	2B	183.77
IWA6175	2B	183.77
IWA6969	2B	183.77
IWA1036	2B	184.07
IWA1690	2B	184.07
IWA4853	2B	184.07
IWA5525	2B	184.07
IWA5547	2B	184.07
IWA8359	2B	184.37
Xbarc128	2B	184.95
IWA68	2B	186.12
wPt_3272	2B	186.71
wPt_1140	2B	188.26
IWA4636	2B	188.26
IWA1489	2B	188.26
wPt_2327	2B	188.26
IWA2261	2B	188.89
IWA2903	2B	188.89
IWA833	2B	188.89
IWA2130	2B	188.89
IWA4956	2B	188.89

IWA6093	2B	188.89
IWA8478	2B	189.18
IWA7850	2B	189.18
IWA4358	2B	189.18
IWA933	2B	189.18
IWA3935	2B	189.18
IWA1389	2B	189.18
IWA4357	2B	189.18
IWA4890	2B	189.18
wPt_741721	2B	189.77
wPt_7200	2B	191.94
wPt_2854	2B	191.94
wPt_0950	2B	191.94
IWA1707	2B	192.55
wPt_9654	2B	192.86
wPt_0189	2B	192.86
wPt_1294	2B	192.86
IWA4096	2B	195.09
IWA4098	2B	195.09
IWA4097	2B	195.09
IWA4095	2B	195.41
IWA7371	2B	195.41
IWA7652	2B	196.28
IWA2379	2B	196.6
IWA2701	2B	196.6
IWA2677	2B	196.6
IWA2678	2B	196.6
IWA2676	2B	196.6
IWA3148	2B	196.6
wPt_7305	2B	197.86
IWA3175	2B	198.77
IWA3176	2B	198.77
IWA2872	2B	198.77
IWA6561	2B	199.09
IWA2874	2B	199.09
IWA2873	2B	199.09
IWA1599	2B	200.36
IWA5460	2B	200.36
IWA3010	2B	200.36
IWA8449	2B	200.68
IWA1822	2B	200.68
wPt_0697	2B	201
wPt_4133	2B	201.91
wPt_9257	2B	201.91
wPt_0473	2B	201.91
wPt_3383	2B	201.91
IWA2502	2B	202.79

IWA8266	2B	202.79
IWA1076	2B	203.1
IWA4130	2B	203.1
IWA7955	2B	203.41
IWA1765	2B	203.41
IWA2459	2B	203.41
IWA5024	2B	203.74
Xbarc332	2B	204.74
wPt_0694	2B	205.03
wPt_3755	2B	205.03
wPt_745051	2B	205.33
wPt_0510	2B	207.62
wPt_666931	2B	210.59
wPt_9190	2B	211.83
tPt_9767	2B	213.05
wPt_7004	2B	213.35
wPt_7161	2B	213.65
wPt_2415	2B	216.05
wPt_9336	2B	218.39
wPt_3898	2B	219.66
wPt_7350	2B	220.62
wPt_741382	2B	221.58
IWA5270	2B	224.79
IWA5081	2B	225.39
IWA8589	2B	226.63
IWA571	2B	227.83
IWA570	2B	227.83
wPt_732040	2B	235.83
wPt_0471	2B	235.83
IWA3474	2B	243.29
IWA8055	2B	248.99
IWA746	2B	248.99
IWA2094	2B	255.65
wPt_2135	2B	256.29
wPt_3378	2B	256.29
wPt_7360	2B	256.89
wPt_2274	2B	256.89
wPt_5736	2B	257.8
IWA5694	2B	257.8
IWA6852	2B	257.8
IWA2946	2B	258.5
wPt_4773	2B	259.95
wPt_4368	2B	261.88
Xbarc159	2B	262.79
IWA2046	2B	263.83
wPt_2724	2B	264.52
wPt_6643	2B	265.14

	Xgwm382a	2B	266.99
	IWA2551	2B	270.03
	IWA8018	2B	271.06
	IWA1667	2B	271.4
	IWA3252	2B	271.75
	wPt_8916	2B	272.44
	wPt_7322	2B	275.13
**Group 7**	wPt_9997	2D	0
	Ppd_D1	2D	9.56
	Xgwm484	2D	20.28
	IWA4496	2D	30.97
	Xgwm102	2D	33.24
	IWA6374	2D	35.66
	IWA209	2D	37.01
	wPt_733567	2D	40.56
	wPt_731409	2D	42.19
	wPt_667312	2D	42.19
	wPt_666987	2D	44.33
	wPt_4144	2D	45.36
	wPt_740836	2D	45.66
	wPt_730889	2D	47.21
	wPt_734280	2D	47.21
	wPt_665644	2D	47.83
	IWA144	2D	48.43
	IWA8151	2D	54.09
	wPt_742606	2D	55.99
	wPt_2761	2D	56.59
	wPt_665342	2D	56.59
	IWA5637	2D	57.51
	IWA2961	2D	57.51
	IWA8562	2D	57.51
	wPt_732942	2D	71.03
	wPt_2544	2D	88.52
**Group 8**	wPt_2938	3A	0
	wPt_10311	3A	0.68
	wPt_4868	3A	2.31
	IWA2993	3A	4.32
	IWA7861	3A	4.32
	wPt_740544	3A	5.27
	wPt_8855	3A	5.56
	wPt_2748	3A	5.56
	wPt_671711	3A	5.56
	wPt_741848	3A	5.56
	wPt_743909	3A	5.56
	wPt_732029	3A	5.56
	wPt_741816	3A	5.56
	wPt_741986	3A	5.56

wPt_742118	3A	5.56
wPt_742665	3A	5.56
wPt_742486	3A	5.56
wPt_743858	3A	5.56
wPt_741078	3A	5.56
wPt_741976	3A	5.56
wPt_7992	3A	6.15
tPt_0519	3A	6.15
wPt_6854	3A	6.15
wPt_1111	3A	6.15
tPt_6949	3A	6.15
IWA1269	3A	7.1
IWA1270	3A	7.1
IWA5428	3A	7.1
IWA8587	3A	7.1
IWA2737	3A	7.1
wPt_1655	3A	7.69
IWA2738	3A	8.58
IWA8280	3A	8.58
IWA8127	3A	10.55
wPt_1939	3A	11.79
IWA5969	3A	12.78
Xwmc532	3A	13.74
wPt_0714	3A	20.57
IWA4257	3A	22.76
IWA4781	3A	23.09
IWA5050	3A	23.09
IWA5642	3A	46.91
IWA3739	3A	46.91
IWA3448	3A	47.22
IWA7073	3A	65.39
IWA1294	3A	72.64
IWA6997	3A	72.64
IWA6996	3A	72.97
IWA2348	3A	75.04
IWA5114	3A	75.04
IWA2362	3A	79.98
IWA1611	3A	79.98
IWA2028	3A	83.51
IWA2029	3A	83.51
IWA523	3A	83.82
IWA524	3A	83.82
IWA799	3A	83.82
IWA926	3A	83.82
IWA5456	3A	88.37
IWA4810	3A	88.37
IWA1778	3A	88.37

	IWA2958	3A	88.37
	IWA3524	3A	88.37
	IWA4296	3A	88.37
	IWA4297	3A	88.37
	IWA4298	3A	88.37
	IWA4308	3A	88.37
	IWA5455	3A	88.37
	IWA6055	3A	88.37
	IWA6174	3A	88.37
	IWA7469	3A	88.37
	IWA7643	3A	88.37
	IWA7695	3A	88.37
	IWA7696	3A	88.37
	wPt_732716	3A	95.46
	wPt_2127	3A	113.29
	wPt_8699	3A	116.23
	IWA2518	3A	119.98
	IWA1366	3A	153.02
	IWA1367	3A	153.02
	IWA7938	3A	153.34
	IWA7812	3A	154.86
	IWA1673	3A	157.68
	tPt_7492	3A	157.68
	wPt_9761	3A	157.68
	tPt_9901	3A	157.68
	wPt_4128	3A	157.68
	wPt_743015	3A	157.68
	wPt_9268	3A	159.23
	IWA3559	3A	166.58
	IWA3560	3A	166.58
	IWA5112	3A	169.27
	IWA2397	3A	171.43
	IWA7999	3A	171.43
	IWA5190	3A	171.75
	IWA5191	3A	171.75
	IWA2396	3A	172.08
	IWA8000	3A	172.39
	IWA6716	3A	172.39
	wPt_5476	3A	174.73
	IWA6951	3A	179.88
**Group 9**	Xgwm389	3B	0
	wPt_11419	3B	1.55
	IWA194	3B	4.42
	IWA6587	3B	5.36
	wPt_6043	3B	7.79
	wPt_3260	3B	8.77
	wPt_744251	3B	9.74

wPt_7984	3B	10.74
wPt_10192	3B	12.8
IWA4654	3B	13.79
IWA6471	3B	14.48
IWA5202	3B	14.48
IWA3103	3B	14.48
wPt_2557	3B	16.56
wPt_10710	3B	19.85
tPt_9267	3B	23.03
IWA3724	3B	24.1
IWA6651	3B	24.1
Xbarc133	3B	25.73
IWA4801	3B	28.51
IWA4800	3B	28.51
IWA7174	3B	28.86
IWA5106	3B	28.86
IWA5426	3B	28.86
IWA5299	3B	29.83
IWA5347	3B	29.83
wPt_742528	3B	34.29
wPt_742648	3B	35.61
Xgwm493	3B	38.93
wPt_10647	3B	41.08
wPt_3038	3B	42.33
wPt_6145	3B	42.33
wPt_7212	3B	45.62
wPt_666318	3B	60.57
wPt_11261	3B	61.22
wPt_8238	3B	67.8
IWA8457	3B	82.31
IWA2074	3B	82.31
IWA1414	3B	82.31
IWA1416	3B	82.31
IWA1417	3B	82.31
IWA6919	3B	82.61
IWA6920	3B	82.61
IWA347	3B	82.61
IWA4575	3B	82.61
IWA3149	3B	82.91
IWA3150	3B	82.91
IWA3726	3B	82.91
IWA1458	3B	82.91
IWA4838	3B	82.91
IWA4843	3B	82.91
IWA6482	3B	82.91
IWA2119	3B	83.25
IWA7748	3B	83.25

Xgwm566	3B	83.83
wPt_0371	3B	84.42
rPt_8896	3B	84.72
wPt_732457	3B	85.66
wPt_7486	3B	85.97
IWA5325	3B	86.61
wPt_9579	3B	88.87
wPt_2936	3B	88.87
wPt_6216	3B	88.87
wPt_3046	3B	88.87
wPt_730063	3B	90.44
wPt_1612	3B	93.11
IWA3426	3B	94.82
IWA6632	3B	94.82
IWA3425	3B	94.82
IWA292	3B	98.5
IWA5677	3B	98.5
IWA210	3B	99.13
IWA5787	3B	99.13
IWA5788	3B	99.13
IWA5813	3B	99.13
IWA6014	3B	99.13
IWA6698	3B	99.13
IWA729	3B	99.46
IWA1206	3B	99.46
IWA5351	3B	99.46
IWA5638	3B	99.46
rPt_5396	3B	100.71
wPt_743661	3B	100.71
wPt_3726	3B	101.01
wPt_6905	3B	102.85
IWA2492	3B	104.17
wPt_8915	3B	105.14
wPt_0327	3B	105.14
IWA6655	3B	106.41
IWA5770	3B	106.73
IWA3021	3B	106.73
IWA4235	3B	107.72
IWA4236	3B	107.72
IWA6297	3B	108.39
IWA4613	3B	108.72
IWA3245	3B	109.05
IWA5101	3B	109.36
IWA2022	3B	109.36
IWA7510	3B	109.36
IWA8196	3B	109.7
IWA3710	3B	109.7

IWA4439	3B	109.7
IWA5100	3B	109.7
IWA7519	3B	109.99
IWA4206	3B	110.56
IWA4267	3B	110.56
IWA4269	3B	110.56
IWA7236	3B	110.56
IWA8180	3B	110.56
IWA1703	3B	110.56
IWA1704	3B	110.56
IWA4721	3B	110.9
IWA5178	3B	110.9
IWA7516	3B	110.9
IWA3304	3B	112.24
IWA5775	3B	112.24
IWA6492	3B	112.55
IWA7512	3B	112.55
IWA3306	3B	112.55
IWA4653	3B	112.55
IWA6221	3B	114.94
IWA2399	3B	117.72
IWA2510	3B	117.72
IWA1598	3B	117.72
IWA2400	3B	117.72
IWA2124	3B	118.07
IWA3402	3B	118.4
IWA3601	3B	118.4
IWA7125	3B	121.44
rPt_7068	3B	122.05
wPt_2280	3B	123.63
wPt_1171	3B	124.25
IWA7889	3B	124.56
IWA3591	3B	124.56
IWA5646	3B	124.56
IWA8249	3B	124.56
IWA3593	3B	124.56
IWA2619	3B	124.56
IWA2620	3B	124.56
IWA7819	3B	124.56
IWA7888	3B	124.56
IWA1769	3B	124.56
wPt_7688	3B	124.56
IWA8104	3B	124.87
IWA8086	3B	124.87
IWA1576	3B	124.87
IWA1095	3B	124.87
IWA2951	3B	124.87

IWA3455	3B	124.87
IWA3592	3B	124.87
IWA5985	3B	124.87
IWA7108	3B	125.16
IWA7391	3B	125.16
IWA8490	3B	125.74
IWA5984	3B	125.74
IWA7680	3B	126.06
IWA3274	3B	126.36
IWA7679	3B	126.66
IWA5510	3B	127.87
IWA6002	3B	127.87
IWA2978	3B	128.16
IWA4146	3B	128.16
IWA6513	3B	128.16
IWA5511	3B	128.16
IWA3170	3B	128.16
Xgwm108	3B	129.67
IWA2462	3B	130.31
IWA5013	3B	130.62
IWA4324	3B	131.26
IWA4778	3B	132.2
IWA6056	3B	132.2
wPt_8056	3B	134.15
wPt_0065	3B	135.37
wPt_10384	3B	138.44
IWA4600	3B	140.55
wPt_664981	3B	141.51
wPt_672088	3B	141.51
wPt_5769	3B	141.51
wPt_5358	3B	141.51
wPt_2904	3B	142.12
wPt_7968	3B	143.09
wPt_3678	3B	143.72
wPt_10179	3B	150.81
wPt_10170	3B	151.73
IWA1094	3B	153.95
wPt_5704	3B	168.4
IWA939	3B	171.95
wPt_11029	3B	177.23
wPt_0021	3B	177.23
wPt_7037	3B	184.06
wPt_667746	3B	185.04
wPt_5261	3B	185.04
IWA6930	3B	185.97
wPt_0367	3B	188.93
wPt_10537	3B	189.23

	IWA6273	3B	191.78
	wPt_3342	3B	191.78
	wPt_729962	3B	191.78
	wPt_0900	3B	191.78
	wPt_742794	3B	191.78
	wPt_9342	3B	191.78
	IWA8185	3B	192.11
	wPt_666728	3B	192.75
	wPt_9368	3B	194.96
	wPt_0912	3B	194.96
	IWA4311	3B	195.27
	IWA4312	3B	195.27
	wPt_3541	3B	195.27
	wPt_8845	3B	202.2
	wPt_8206	3B	202.51
	wPt_10758	3B	207.16
	wPt_6834	3B	207.16
	wPt_743330	3B	211.69
	Xgwm247	3B	218.4
	wPt_10071	3B	220.29
	wPt_3856	3B	220.6
**Group 10**	Xgwm52	3D	0
	IWA1321	3D	0.31
	IWA4559	3D	0.31
	IWA5695	3D	0.31
	Xgdm8	3D	1.24
	IWA7468	3D	1.53
	Xbarc125	3D	2.41
	Xcfd4b	3D	4.21
	Xgwm456	3D	5.69
	IWA6119	3D	8.42
	IWA7672	3D	8.42
	Xcfd70	3D	8.42
	wPt_672034	3D	10.55
	wPt_732092	3D	12.77
	Xgdm72	3D	19.13
	wPt_742488	3D	33.83
	wPt_740798	3D	33.83
	wPt_742689	3D	33.83
	wPt_5215	3D	33.83
	wPt_4476	3D	33.83
	wPt_740930	3D	33.83
	wPt_742242	3D	33.83
	wPt_671740	3D	33.83
	wPt_742576	3D	33.83
	wPt_742151	3D	33.83
	wPt_742363	3D	33.83

	wPt_742360	3D	33.83
	wPt_742810	3D	33.83
	wPt_740584	3D	33.83
	wPt_742491	3D	33.83
	wPt_740598	3D	33.83
	wPt_741446	3D	33.83
	wPt_741128	3D	33.83
	wPt_6066	3D	35.87
	wPt_740564	3D	37.66
**Group 11**	Xbarc52	4A	0
	wPt_4064	4A	3.11
	wPt_9675	4A	3.44
	wPt_2792	4A	4.06
	wPt_0764	4A	5.3
	wPt_3402	4A	5.3
	wPt_734161	4A	5.3
	wPt_666456	4A	5.3
	IWA4030	4A	6.18
	IWA3422	4A	6.18
	IWA1835	4A	6.18
	IWA1836	4A	6.18
	IWA7305	4A	6.18
	IWA7197	4A	6.18
	IWA1674	4A	6.18
	IWA6392	4A	6.18
	wPt_1155	4A	6.18
	wPt_4424	4A	6.18
	wPt_5578	4A	6.18
	wPt_9418	4A	6.18
	wPt_5434	4A	6.18
	wPt_1007	4A	6.18
	IWA7304	4A	6.46
	IWA2756	4A	6.46
	IWA3449	4A	6.46
	IWA4859	4A	8.24
	IWA7364	4A	8.24
	IWA1410	4A	8.24
	IWA4084	4A	8.24
	IWA4858	4A	8.24
	IWA5353	4A	8.24
	IWA7365	4A	8.24
	wPt_11295	4A	10.71
	IWA4083	4A	12.89
	wPt_2951	4A	13.19
	wPt_0150	4A	13.48
	IWA4689	4A	13.79
	IWA4688	4A	13.79

wPt_6688	4A	14.38
wPt_3250	4A	14.38
wPt_8091	4A	14.38
wPt_2533	4A	14.38
wPt_1161	4A	14.38
wPt_4680	4A	14.68
wPt_6757	4A	14.68
wPt_4828	4A	16.3
wPt_0023	4A	25.94
IWA558	4A	55.33
IWA559	4A	55.64
wPt_4645	4A	56.91
wPt_9833	4A	56.91
wPt_8517	4A	61.37
Xbarc343	4A	64.71
Xwmc262	4A	65
wPt_5857	4A	66.82
IWA7322	4A	68.64
IWA811	4A	68.64
IWA3698	4A	68.96
IWA3061	4A	68.96
IWA485	4A	70.25
IWA3188	4A	70.25
IWA5152	4A	70.25
IWA8168	4A	70.85
IWA6418	4A	71.15
IWA3774	4A	72.19
Xwmc161	4A	78.18
Xwmc707	4A	84.71
IWA3756	4A	87.11
IWA3757	4A	87.45
IWA3758	4A	87.45
IWA7118	4A	92.44
IWA2904	4A	92.74
IWA6906	4A	93.09
IWA2106	4A	94.9
IWA5477	4A	94.9
IWA1692	4A	95.6
IWA1691	4A	95.95
IWA1529	4A	95.95
IWA5116	4A	97.35
IWA1720	4A	97.7
IWA5200	4A	97.7
Xgwm397	4A	105.25
IWA2460	4A	114.31
tPt_9856	4A	125.79
tPt_4753	4A	125.79

	wPt_9778	4A	126.1
	wPt_741979	4A	126.42
	Xwmc48	4A	140.33
	IWA482	4A	147.57
	IWA6501	4A	147.57
	IWA2793	4A	147.57
	IWA5544	4A	147.57
	IWA4867	4A	147.57
	IWA7448	4A	147.57
	IWA4698	4A	147.57
	IWA5123	4A	147.57
	wPt_3515	4A	147.57
	IWA2794	4A	148.2
	IWA402	4A	148.2
	IWA1792	4A	148.2
	IWA5699	4A	148.2
	IWA3191	4A	148.52
	IWA7699	4A	148.52
	IWA2585	4A	148.84
	IWA2900	4A	148.84
	IWA2901	4A	148.84
	IWA3584	4A	152.37
	IWA4261	4A	153.69
	IWA4260	4A	154.02
	IWA4320	4A	154.67
	IWA4321	4A	154.99
	IWA5363	4A	155.65
	IWA1137	4A	157.39
	IWA7394	4A	160.41
	IWA2764	4A	162.79
**Group 12**	Xwmc141	4B	0
	IWA506	4B	1.78
	IWA1768	4B	2.09
	IWA7266	4B	8.18
	IWA2125	4B	8.18
	IWA8178	4B	8.48
	Xmag4087	4B	11.8
	Xmag2055	4B	13.61
	Xwmc710	4B	14.78
	IWA1045	4B	16.04
	IWA4662	4B	17.03
	IWA2194	4B	22.69
	Xswes24	4B	25.08
	Xgpw7026	4B	26.56
	Xwmc657	4B	31.68
	Xwmc695	4B	36.12
	Xbarc20	4B	37.6

IWA4854	4B	40.36
IWA2313	4B	40.7
Xgdm61	4B	44.2
Xgwm495	4B	49.25
IWA6480	4B	50.51
IWA7462	4B	51.48
IWA1641	4B	51.48
IWA6828	4B	51.48
IWA2218	4B	51.48
IWA3287	4B	51.48
IWA7900	4B	51.48
IWA7461	4B	51.78
Xgwm251	4B	52.36
IWA7463	4B	52.97
IWA6827	4B	52.97
IWA3610	4B	53.63
IWA3611	4B	53.63
IWA4640	4B	53.63
wPt_733038	4B	54.28
wPt_6209	4B	54.28
IWA187	4B	54.86
IWA3551	4B	54.86
IWA3608	4B	54.86
IWA3609	4B	54.86
IWA1382	4B	55.19
IWA2393	4B	55.19
IWA2755	4B	55.19
IWA2974	4B	55.19
IWA2171	4B	55.5
IWA2754	4B	55.5
IWA2823	4B	55.82
Xbarc163	4B	56.12
Xcfd22	4B	56.71
Xgpw7272	4B	57.3
Xcfd39	4B	59.41
Xgpw7390	4B	62.79
wPt_744934	4B	64.74
IWA1113	4B	66.75
IWA3038	4B	70.18
IWA3041	4B	70.18
IWA3042	4B	70.18
IWA3039	4B	70.51
Xgwm6	4B	73.18
IWA3279	4B	77.8
Xgpw4175	4B	78.41
IWA3697	4B	83.85
Xbarc114	4B	87.54

	IWA5358	4B	91.04
	IWA564	4B	91.66
	IWA3781	4B	91.97
	IWA27	4B	92.89
	wPt_731869	4B	94.99
	wPt_3917	4B	94.99
	wPt_730303	4B	94.99
**Group 13**	Xgwm194	4D	0
	Xgwm624	4D	12.77
	Xgwm609	4D	13.35
**Group 14**	IWA2163	5A1	0
	IWA1258	5A1	2.34
	IWA3623	5A1	6.65
	IWA2959	5A1	6.65
	IWA4744	5A1	8.56
	IWA7911	5A1	12.91
	IWA4394	5A1	12.91
	IWA4805	5A1	14.12
	Vrn_A1	5A1	16.93
	IWA6682	5A1	17.52
	IWA3085	5A1	17.81
	IWA1752	5A1	18.4
	IWA6961	5A1	18.7
	IWA7579	5A1	18.7
**Group 15**	wPt_666357	5A2	0
	wPt_742000	5A2	0.3
	IWA7351	5A2	15.36
	wPt_1954	5A2	15.97
	wPt_9452	5A2	16.59
	wPt_3620	5A2	16.59
	tPt_9702	5A2	16.59
	wPt_3884	5A2	16.59
	IWA154	5A2	17.19
	IWA3445	5A2	17.19
	IWA4736	5A2	17.51
	Xbarc180	5A2	17.83
	IWA5539	5A2	19.06
	IWA7130	5A2	19.06
	IWA7129	5A2	19.36
	IWA7598	5A2	19.36
	IWA5033	5A2	21.08
	IWA5032	5A2	21.41
	IWA5034	5A2	21.41
	IWA3873	5A2	22.02
	Xbarc100	5A2	22.02
	IWA6255	5A2	28.35
	IWA3646	5A2	29.03

IWA3645	5A2	29.03
IWA7742	5A2	29.03
IWA3283	5A2	29.03
IWA4237	5A2	29.38
IWA3313	5A2	30.46
IWA4670	5A2	30.82
IWA1686	5A2	31.49
IWA1685	5A2	31.82
IWA8559	5A2	31.82
IWA6949	5A2	32.13
IWA6574	5A2	33.48
IWA1236	5A2	35.87
IWA3717	5A2	37.89
IWA3827	5A2	39.56
wPt_3563	5A2	41.2
Xgwm264	5A2	46.42
wPt_9748	5A2	50.89
IWA6523	5A2	53.1
IWA6522	5A2	53.1
IWA5567	5A2	58.38
IWA4048	5A2	59.38
IWA5529	5A2	60.72
Xgwm156	5A2	62.05
Xbarc40	5A2	62.63
wPt_7769	5A2	64.16
wPt_742925	5A2	64.8
Xcfa2250	5A2	76.37
IWA333	5A2	83.07
wPt_798342	5A2	93.68
wPt_797382	5A2	95.25
wPt_798198	5A2	96.81
wPt_797750	5A2	98.08
wPt_798459	5A2	99.36
wPt_797380	5A2	99.36
wPt_798702	5A2	99.36
wPt_667213	5A2	99.36
wPt_797479	5A2	100.3
wPt_798339	5A2	101.87
wPt_798280	5A2	103.45
wPt_798386	5A2	105.42
wPt_4131	5A2	109.94
IWA6859	5A2	117.61
IWA1568	5A2	122.84
Xgwm443	5A2	124.38
wPt_741087	5A2	126.6
IWA1569	5A2	126.94
IWA3197	5A2	129.35

	IWA6463	5A2	129.35
	IWA3196	5A2	129.66
	IWA4871	5A2	141.55
	Xbarc10	5A2	146.53
	IWA3566	5A2	149.29
	IWA5924	5A2	149.29
	IWA3567	5A2	149.29
	IWA3568	5A2	149.29
	IWA5923	5A2	149.29
	IWA480	5A2	154.73
	IWA2144	5A2	154.73
	IWA2146	5A2	154.73
	IWA2143	5A2	155.02
	IWA2145	5A2	155.02
	wPt_741613	5A2	173
**Group 16**	IWA6580	5B	0
	IWA2682	5B	0.33
	IWA6577	5B	0.33
	IWA6578	5B	0.33
	IWA2238	5B	2.31
	wPt_4986	5B	8.14
	wPt_7665	5B	10.75
	tPt_3621	5B	11.96
	IWA4329	5B	14.72
	IWA5803	5B	14.72
	IWA7701	5B	14.72
	IWA8391	5B	14.72
	IWA8006	5B	14.72
	IWA4903	5B	15.04
	IWA5802	5B	15.04
	wPt_2804	5B	17.36
	wPt_730313	5B	17.36
	wPt_8449	5B	28.87
	wPt_1348	5B	28.87
	Xbarc243	5B	33.02
	IWA6902	5B	45.06
	Xcfd10	5B	48.84
	Xwmc640c	5B	56.08
	Xwmc640a	5B	69.33
	wPt_8872	5B	82.5
	wPt_9504	5B	82.82
	IWA3214	5B	84.08
	IWA3984	5B	84.4
	wPt_2812	5B	89.52
	IWA7340	5B	95.18
	IWA8031	5B	95.18
	IWA6416	5B	96.52

IWA2827	5B	98.24
IWA6097	5B	98.24
IWA8433	5B	98.24
wPt_6880	5B	99.54
wPt_0921	5B	99.54
wPt_743189	5B	99.54
IWA7020	5B	100.42
IWA8444	5B	100.42
IWA4763	5B	103.33
IWA2659	5B	103.33
IWA4762	5B	103.66
IWA3394	5B	103.98
IWA4162	5B	104.3
IWA7393	5B	104.95
IWA1433	5B	104.95
IWA6148	5B	104.95
IWA6779	5B	104.95
IWA7493	5B	104.95
IWA7494	5B	104.95
IWA4182	5B	105.25
IWA4184	5B	105.25
IWA4185	5B	105.25
IWA6147	5B	105.25
tPt_1253	5B	106.24
Xbarc232	5B	106.83
Xbarc266	5B	110.21
Xgdm116	5B	112.94
Xbarc142	5B	118.02
wPt_8094	5B	121.13
wPt_0935	5B	121.13
wPt_9205	5B	121.13
IWA5179	5B	124.22
IWA7668	5B	124.22
IWA8262	5B	124.22
IWA7963	5B	124.22
IWA2500	5B	124.55
IWA7791	5B	124.55
Xgwm604	5B	126.34
IWA1460	5B	128.71
IWA3800	5B	129.05
IWA7732	5B	131.53
Xgwm408	5B	135.97
Vrn_B1	5B	142.83
IWA8395	5B	144.61
IWA5950	5B	145.25
IWA3479	5B	145.6
IWA2565	5B	145.6

IWA6024	5B	145.6
IWA1774	5B	145.95
IWA3002	5B	149.06
IWA3444	5B	150.5
IWA8097	5B	151.18
IWA4566	5B	154.71
IWA8080	5B	161.2
IWA2729	5B	161.51
IWA3643	5B	162.47
IWA3640	5B	162.47
IWA3641	5B	162.47
IWA3644	5B	162.47
IWA3642	5B	162.78
Xcfd7	5B	165.36
wPt_3661	5B	167.86
IWA7175	5B	180.83
wPt_1409	5B	181.73
IWA4832	5B	182.68
IWA337	5B	182.68
IWA987	5B	182.68
IWA2694	5B	182.68
IWA2695	5B	182.68
IWA3964	5B	182.68
IWA4641	5B	183
IWA338	5B	183
IWA7795	5B	183.66
IWA265	5B	184
IWA2455	5B	187.27
wPt_3012	5B	189.99
wPt_8163	5B	189.99
tPt_7755	5B	189.99
wPt_5514	5B	189.99
wPt_742141	5B	189.99
IWA3436	5B	191.04
IWA2432	5B	191.37
IWA4622	5B	191.37
IWA5217	5B	191.37
IWA2536	5B	191.37
IWA2934	5B	191.37
IWA6291	5B	191.37
IWA5331	5B	191.37
IWA5485	5B	191.37
IWA5486	5B	191.37
IWA6638	5B	191.37
IWA4958	5B	192.05
IWA3985	5B	192.73
IWA6867	5B	192.73

IWA6383	5B	193.04
IWA822	5B	193.04
IWA3025	5B	193.04
IWA7636	5B	193.04
IWA4346	5B	193.64
IWA7165	5B	193.64
IWA1471	5B	197.74
IWA7123	5B	197.74
IWA6992	5B	198.75
IWA721	5B	199.44
IWA5743	5B	199.76
IWA3633	5B	199.76
IWA8603	5B	199.76
IWA303	5B	200.38
IWA2003	5B	200.69
IWA1585	5B	201
IWA6689	5B	201
IWA8518	5B	201
IWA5280	5B	201
IWA1584	5B	201.32
IWA5289	5B	201.32
IWA7127	5B	201.32
IWA302	5B	201.32
IWA1777	5B	201.32
IWA5279	5B	201.32
IWA6344	5B	201.32
IWA396	5B	201.94
IWA1706	5B	201.94
IWA2071	5B	201.94
IWA1705	5B	202.26
IWA4526	5B	203.54
IWA6846	5B	203.54
IWA468	5B	203.54
IWA7613	5B	203.54
IWA3682	5B	203.84
IWA7953	5B	203.84
IWA1057	5B	203.84
IWA6030	5B	203.84
IWA3870	5B	204.16
IWA1781	5B	204.16
IWA279	5B	204.16
IWA6773	5B	204.45
IWA2563	5B	205.06
IWA6067	5B	205.06
IWA3101	5B	205.06
IWA6065	5B	205.06
Xgwm544	5B	205.67

wPt_741933	5B	205.96
Xbarc4	5B	208.1
Xgwm540	5B	208.97
IWA5079	5B	209.9
IWA4379	5B	209.9
IWA5078	5B	209.9
IWA4377	5B	210.23
IWA4378	5B	210.23
IWA1965	5B	210.56
IWA1084	5B	210.56
IWA1342	5B	210.56
IWA1394	5B	210.56
IWA3209	5B	210.56
IWA7609	5B	210.89
IWA7608	5B	211.19
Xbarc109	5B	213.59
tPt_8942	5B	217.69
wPt_4736	5B	217.69
wPt_2041	5B	220.57
Xwmc740	5B	221.79
IWA2609	5B	223.73
IWA2610	5B	224.07
IWA3032	5B	227.31
IWA6946	5B	227.31
IWA3033	5B	227.63
IWA4856	5B	227.63
IWA1144	5B	227.63
IWA6947	5B	227.63
wPt_7029	5B	230.76
wPt_5120	5B	231.38
wPt_5175	5B	234.53
wPt_5346	5B	234.53
wPt_665267	5B	234.53
wPt_0208	5B	235.78
wPt_6348	5B	236.73
Xgdm152	5B	237.66
wPt_1784	5B	245.12
wPt_8132	5B	250.14
wPt_9814	5B	250.14
wPt_5737	5B	250.14
tPt_0228	5B	250.14
wPt_666323	5B	262.81
wPt_9006	5B	273.94
wPt_3055	5B	277.49
IWA3457	5B	283.15
IWA5536	5B	283.15
IWA5537	5B	283.15

	IWA7400	5B	283.15
	IWA4416	5B	283.47
	IWA4790	5B	283.78
	IWA4415	5B	283.78
**Group 17**	Xgwm174	5D	0
	Xgdm43	5D	4.73
	Xgwm583	5D	13.55
	wPt_665999	5D	17.28
	Xcfd8	5D	22.39
	wPt_0886	5D	35.46
	wPt_666070	5D	39.47
	IWA5012	5D	45.32
	IWA4550	5D	46.89
	IWA1172	5D	46.89
	IWA5366	5D	47.85
	IWA6052	5D	48.49
	IWA7243	5D	48.49
	IWA7517	5D	48.49
	Xcfd78	5D	51.36
	rPt_3825	5D	90.16
	Xgwm654	5D	92.62
	Xwmc443	5D	93.79
	Xbarc177	5D	106.09
	Xbarc161	5D	106.09
	Xwmc765	5D	106.09
	IWA700	5D	106.38
	IWA701	5D	106.38
	IWA6060	5D	106.38
	IWA6061	5D	106.38
	IWA699	5D	106.68
	IWA6059	5D	106.68
	IWA6190	5D	107.62
	IWA4293	5D	107.95
	IWA6189	5D	108.27
	IWA2821	5D	109.53
	IWA5532	5D	111.5
	Xwmc640b	5D	121.11
	wPt_733708	5D	144.46
	wPt_667042	5D	144.77
	wPt_667461	5D	144.77
	wPt_664920	5D	150.66
	Xgwm212	5D	159.99
	IWA4087	5D	174.62
	wPt_5505	5D	175.27
	wPt_1400	5D	175.56
**Group 18**	Xcfd1b	6A	0
	Xbarc23	6A	19.43

IWA6013	6A	27.29
IWA647	6A	27.29
IWA1335	6A	27.29
IWA1338	6A	27.29
IWA2137	6A	27.62
Xcfd1c	6A	28.56
IWA8608	6A	29.44
wPt_9132	6A	30.03
wPt_732760	6A	30.32
wPt_3965	6A	30.32
wPt_9113	6A	30.32
wPt_7623	6A	30.32
wPt_731010	6A	30.62
wPt_734004	6A	31.21
wPt_730631	6A	31.21
wPt_665782	6A	31.21
wPt_667170	6A	31.84
wPt_0959	6A	34.93
wPt_734068	6A	34.93
IWA4961	6A	36.47
IWA6630	6A	36.47
IWA1336	6A	36.47
IWA1522	6A	36.47
IWA1523	6A	36.47
wPt_730729	6A	36.47
wPt_2573	6A	36.47
wPt_666988	6A	36.47
wPt_9692	6A	36.47
tPt_6278	6A	36.47
wPt_2822	6A	37.97
wPt_9584	6A	37.97
wPt_667780	6A	37.97
wPt_664733	6A	37.97
wPt_733764	6A	37.97
wPt_665036	6A	37.97
IWA770	6A	38.85
Xcfd1a	6A	39.44
IWA6390	6A	40.06
IWA1282	6A	40.06
IWA1283	6A	40.06
IWA7286	6A	40.06
IWA1205	6A	44.75
wPt_671855	6A	47.63
tPt_0877	6A	48.64
wPt_7840	6A	49.94
Xgwm334	6A	53.34
wPt_9075	6A	57.73

Xgwm459	6A	58.61
rPt_4564	6A	60.17
IWA7913	6A	62.39
IWA273	6A	64.47
wPt_9832	6A	65.08
wPt_665021	6A	67.1
wPt_0228	6A	69.07
wPt_9382	6A	70.33
wPt_9687	6A	72.64
wPt_1285	6A	73.26
IWA2635	6A	73.98
IWA2413	6A	74.32
IWA6711	6A	74.32
IWA7612	6A	74.32
IWA8160	6A	76.23
wPt_734344	6A	77.44
wPt_8443	6A	78.35
wPt_7475	6A	78.98
wPt_667740	6A	79.29
wPt_0562	6A	79.58
wPt_5964	6A	79.58
wPt_8006	6A	79.58
wPt_2636	6A	79.58
wPt_0864	6A	79.58
wPt_730591	6A	79.58
wPt_741170	6A	79.58
wPt_671561	6A	79.58
wPt_664589	6A	79.58
wPt_672029	6A	79.91
wPt_672131	6A	80.25
wPt_2741	6A	81.55
wPt_732691	6A	82.85
wPt_9306	6A	86
IWA7000	6A	92.06
IWA4208	6A	92.06
IWA6999	6A	92.06
IWA1749	6A	92.06
IWA19	6A	117.6
IWA20	6A	117.6
IWA4951	6A	117.6
IWA5523	6A	117.6
IWA4950	6A	117.91
IWA6775	6A	117.91
Xwmc179	6A	118.84
IWA259	6A	120.01
Xgwm570	6A	120.59
IWA260	6A	120.88

Xwmc553	6A	122.05
IWA2241	6A	123.64
IWA654	6A	123.95
IWA8431	6A	123.95
Xgpw2060	6A	127.31
IWA7397	6A	130.67
IWA7575	6A	130.67
IWA5940	6A	130.67
IWA992	6A	130.67
tPt_4209	6A	135.68
wPt_5310	6A	151.93
wPt_733195	6A	151.93
wPt_730769	6A	151.93
wPt_732183	6A	151.93
Xgwm169	6A	164.31
IWA2538	6A	167.94
IWA2539	6A	167.94
IWA5035	6A	168.95
IWA5398	6A	168.95
IWA4478	6A	171.21
IWA8568	6A	171.21
IWA6858	6A	171.55
IWA504	6A	176.75
IWA6117	6A	177.06
IWA5704	6A	177.37
IWA4602	6A	177.67
IWA4603	6A	177.67
IWA6116	6A	177.98
Xcfa2114	6A	182.3
Xgwm617	6A	185.66
Xgwm427	6A	187.75
IWA1098	6A	188.37
IWA6182	6A	188.37
IWA1000	6A	188.68
IWA442	6A	188.68
Xwmc580	6A	188.68
IWA5172	6A	192.94
IWA2567	6A	192.94
IWA5768	6A	192.94
IWA3247	6A	193.26
wPt_5654	6A	195.26
IWA4699	6A	196.83
IWA1391	6A	196.83
IWA2795	6A	197.14
IWA3246	6A	197.45
IWA3203	6A	197.45
IWA3204	6A	197.45

	IWA3205	6A	197.45
	IWA5746	6A	197.76
	IWA5767	6A	197.76
	IWA5974	6A	197.76
	IWA6305	6A	197.76
	IWA6304	6A	197.76
	IWA2603	6A	197.76
	IWA5747	6A	197.76
	IWA2260	6A	198.37
	IWA3918	6A	198.37
	IWA5582	6A	198.37
	IWA2639	6A	198.37
	IWA7386	6A	198.37
	IWA3066	6A	198.37
	IWA1510	6A	198.67
	wPt_731936	6A	199.31
	IWA7894	6A	199.91
	wPt_664504	6A	206.19
	wPt_744786	6A	206.49
	wPt_664937	6A	206.79
	Xwmc621	6A	210.27
**Group 19**	wPt_740675	6B	0
	wPt_6293	6B	12.06
	wPt_6585	6B	13.94
	wPt_5673	6B	14.24
	wPt_4012	6B	15.23
	wPt_734331	6B	16.27
	IWA2479	6B	20.33
	wPt_8894	6B	20.33
	wPt_1547	6B	20.33
	wPt_1756	6B	22.57
	IWA666	6B	22.88
	IWA1901	6B	30.02
	IWA5857	6B	30.34
	IWA5942	6B	30.34
	IWA5943	6B	30.34
	IWA4612	6B	30.34
	IWA7841	6B	30.34
	wPt_732062	6B	31.61
	wPt_733856	6B	31.61
	wPt_1241	6B	40.8
	IWA4290	6B	44.18
	IWA7725	6B	44.18
	wPt_663764	6B	47.21
	IWA52	6B	50.67
	IWA1849	6B	51.25
	IWA2472	6B	51.25

IWA1850	6B	51.61
IWA1851	6B	51.61
wPt_3309	6B	52.91
wPt_5333	6B	53.21
Xwmc487	6B	54.39
wPt_2297	6B	55.29
wPt_1311	6B	56.59
IWA4501	6B	74.4
IWA4502	6B	74.4
IWA755	6B	74.4
IWA971	6B	74.4
IWA5044	6B	74.4
IWA5045	6B	74.4
Xgwm107	6B	75
wPt_4648	6B	75.59
Xgwm193	6B	76.78
wPt_4062	6B	79.07
wPt_3070	6B	80.05
wPt_1364	6B	80.05
wPt_8976	6B	80.05
wPt_9145	6B	80.05
Xbarc198	6B	80.35
wPt_2537	6B	80.95
IWA8539	6B	81.56
IWA1151	6B	81.56
IWA2135	6B	81.56
IWA3167	6B	81.56
IWA4599	6B	81.56
IWA2090	6B	81.56
IWA2244	6B	81.56
IWA3168	6B	81.56
IWA3450	6B	81.56
IWA1815	6B	81.56
IWA5029	6B	81.56
IWA2300	6B	81.56
IWA8191	6B	81.56
IWA8144	6B	81.56
IWA8175	6B	81.56
IWA185	6B	81.56
IWA1545	6B	81.56
IWA2811	6B	81.56
IWA3132	6B	81.56
IWA5096	6B	81.56
IWA5098	6B	81.56
IWA5748	6B	81.56
IWA6142	6B	81.56
IWA6825	6B	81.56

IWA6826	6B	81.56
IWA8189	6B	81.56
IWA2185	6B	81.56
IWA4487	6B	81.56
IWA5104	6B	81.56
IWA5157	6B	81.56
IWA2780	6B	81.56
IWA4924	6B	81.56
IWA1839	6B	81.56
IWA941	6B	81.56
IWA1840	6B	81.56
IWA2843	6B	81.56
IWA5225	6B	81.56
IWA7935	6B	81.56
IWA1742	6B	81.56
IWA1743	6B	81.56
IWA4086	6B	81.56
IWA5242	6B	81.56
IWA5957	6B	81.56
IWA6855	6B	81.56
IWA4169	6B	81.56
IWA6101	6B	81.56
IWA7628	6B	81.56
IWA3030	6B	81.56
Xgwm88	6B	81.56
wPt_4716	6B	81.56
rPt_1040	6B	81.56
wPt_7935	6B	81.56
wPt_2564	6B	81.56
IWA3869	6B	81.84
IWA3878	6B	81.84
IWA3459	6B	81.84
IWA2198	6B	81.84
IWA4783	6B	81.84
IWA7818	6B	81.84
IWA3131	6B	81.84
IWA3133	6B	81.84
IWA3632	6B	81.84
IWA5095	6B	81.84
IWA5531	6B	81.84
IWA5785	6B	81.84
IWA7663	6B	81.84
IWA7873	6B	81.84
IWA8190	6B	81.84
IWA8192	6B	81.84
IWA8184	6B	81.84
IWA2692	6B	81.84

IWA7995	6B	81.84
IWA1838	6B	81.84
IWA7574	6B	81.84
IWA5102	6B	81.84
IWA3652	6B	81.84
IWA617	6B	81.84
IWA7380	6B	81.84
IWA3917	6B	81.84
IWA5241	6B	81.84
IWA5966	6B	81.84
IWA6153	6B	81.84
IWA4170	6B	81.84
IWA5231	6B	81.84
IWA434	6B	81.84
IWA613	6B	81.84
IWA2109	6B	81.84
IWA4848	6B	81.84
IWA6628	6B	81.84
IWA4440	6B	81.84
IWA4500	6B	82.14
IWA4503	6B	82.14
IWA7962	6B	82.14
IWA387	6B	83.06
IWA8611	6B	83.06
IWA683	6B	83.38
wPt_3168	6B	84.68
IWA6428	6B	85.65
IWA4339	6B	85.65
IWA221	6B	85.65
IWA3967	6B	85.97
IWA1472	6B	85.97
IWA7506	6B	86.31
IWA1679	6B	86.66
IWA3636	6B	86.99
IWA4959	6B	86.99
IWA5607	6B	86.99
IWA3327	6B	86.99
IWA4337	6B	86.99
IWA5148	6B	86.99
IWA4338	6B	86.99
Xgwm626	6B	86.99
IWA219	6B	87.29
IWA283	6B	87.29
IWA2773	6B	87.29
IWA2830	6B	87.29
IWA1473	6B	87.29
IWA297	6B	87.29

	IWA4717	6B	107.87
	IWA4717	6B	107.87
	IWA6853	6B	113.87
	IWA6754	6B	116.93
	Xbarc134	6B	117.6
	wPt_1325	6B	127.81
	Xgdm147b	6B	130.56
	Xgdm147a	6B	135.96
	IWA824	6B	139.65
	wPt_4164	6B	141.18
	IWA3224	6B	142.09
	IWA5755	6B	142.09
	wPt_1761	6B	142.09
	wPt_1541	6B	142.09
	IWA4244	6B	142.4
	wPt_9256	6B	142.7
	wPt_6878	6B	143.63
	wPt_744795	6B	143.63
	wPt_3207	6B	143.93
	IWA7098	6B	144.54
	IWA3225	6B	145.18
	IWA4246	6B	145.18
	IWA3222	6B	145.18
	IWA4245	6B	145.18
	IWA8441	6B	145.49
	IWA1233	6B	146.72
	IWA4568	6B	147.66
	wPt_8493	6B	147.96
	wPt_0171	6B	147.96
	wPt_0406	6B	147.96
	wPt_2175	6B	148.56
	wPt_8059	6B	149.45
	wPt_732395	6B	149.45
	Xgpw2344	6B	151.54
**Group 20**	Xcfd49	6D	0
	IWA2965	6D	8.47
	IWA1924	6D	9.44
	IWA3291	6D	10.09
	wPt_731887	6D	24.33
	Xgdm132b	6D	34.83
	Xcfd190	6D	40.99
	wPt_2864	6D	53.18
	wPt_3350	6D	53.18
	wPt_731816	6D	53.18
	wPt_664939	6D	68.88
	Xgdm141	6D	77.1
	Xgwm325	6D	78.58

	wPt_665166	6D	89.51
	IWA6799	6D	111.82
	wPt_664770	6D	112.14
	Xbarc183a	6D	113.09
	wPt_741955	6D	118.29
	wPt_8336	6D	119.87
	IWA6939	6D	121.13
	IWA8060	6D	121.43
	wPt_668152	6D	121.43
	wPt_668181	6D	121.43
	Xgpw5182	6D	123.56
	IWA599	6D	126.83
	wPt_664682	6D	128.75
	Xcfd42	6D	132.49
	IWA7858	6D	136.99
**Group 21**	wPt_0288	7A1	0
	Xbarc222	7A1	2.11
	IWA6331	7A1	2.73
	IWA2042	7A1	4.45
	IWA2820	7A1	4.76
	IWA3754	7A1	4.76
	IWA7731	7A1	4.76
	IWA274	7A1	5.69
	IWA7460	7A1	5.69
	IWA7419	7A1	5.69
	IWA3674	7A1	6.34
	IWA3673	7A1	6.66
	IWA7206	7A1	6.66
	IWA6310	7A1	6.95
	IWA275	7A1	6.95
	IWA5258	7A1	7.29
	wPt_4051	7A1	7.94
	wPt_1080	7A1	7.94
	wPt_7076	7A1	7.94
	Xbarc174	7A1	8.23
	Xwmc826	7A1	20.16
	IWA5904	7A1	38.36
	IWA7005	7A1	38.36
	wPt_6019	7A1	38.36
	Xcfa2257	7A1	39.53
	wPt_1958	7A1	40.41
	IWA7706	7A1	41.59
	IWA8312	7A1	41.59
	IWA7592	7A1	41.59
	IWA6424	7A1	41.59
	IWA6736	7A1	41.91
	IWA7904	7A1	41.91

	IWA5798	7A1	41.91
	IWA5799	7A1	41.91
	Xwmc809	7A1	42.57
	Xgwm344	7A1	44.35
	IWA795	7A1	47.64
	IWA737	7A1	47.98
	Xwmc525	7A1	50.72
	tPt_9518	7A1	51.91
	wPt_3403	7A1	52.5
	wPt_3782	7A1	53.09
	wPt_7122	7A1	53.09
	IWA866	7A1	53.97
	IWA865	7A1	53.97
	IWA4364	7A1	53.97
	IWA761	7A1	54.29
	IWA2929	7A1	54.29
	IWA4028	7A1	54.29
	IWA4173	7A1	54.29
	IWA3371	7A1	54.29
	IWA4175	7A1	54.29
	Xmag1810	7A1	54.59
	IWA4595	7A1	55.89
	IWA7185	7A1	56.22
	IWA6115	7A1	56.22
	IWA4594	7A1	56.22
	IWA4992	7A1	66.76
	IWA4994	7A1	66.76
	IWA6785	7A1	66.76
	wPt_0961	7A1	75.61
	wPt_6620	7A1	75.91
	wPt_4831	7A1	76.21
	IWA7325	7A1	77.81
	Xcfa2019	7A1	81.41
	Xwmc116	7A1	86.49
	IWA4993	7A1	90.81
	IWA4991	7A1	90.81
**Group 22**	Xgwm233	7A2	0
	wPt_0008	7A2	1.56
	wPt_4875	7A2	1.88
	wPt_7608	7A2	3.47
	wPt_8700	7A2	5.42
	wPt_6876	7A2	10.74
	wPt_744818	7A2	14.95
	wPt_664252	7A2	14.95
	wPt_743510	7A2	14.95
	wPt_669103	7A2	14.95
	wPt_4960	7A2	14.95

	wPt_0992	7A2	14.95
	wPt_8149	7A2	14.95
	wPt_7113	7A2	14.95
	wPt_9914	7A2	14.95
	wPt_1093	7A2	14.95
	wPt_4778	7A2	14.95
	wPt_3794	7A2	14.95
	wPt_8043	7A2	14.95
	wPt_6967	7A2	14.95
	wPt_7151	7A2	14.95
	wPt_6417	7A2	16.45
	wPt_8418	7A2	16.45
	wPt_5590	7A2	16.45
	wPt_0744	7A2	16.45
	wPt_7830	7A2	17.67
	tPt_9948	7A2	19.26
	wPt_9496	7A2	20.53
	IWA3850	7A2	21.93
	Xwmc479	7A2	36.3
	wPt_9207	7A2	40.77
	IWA2880	7A2	41.07
	IWA1735	7A2	41.07
	IWA5245	7A2	41.07
	rPt_4199	7A2	41.07
	wPt_5742	7A2	41.07
	wPt_742244	7A2	41.07
	IWA2879	7A2	41.36
	wPt_0040	7A2	41.36
	wPt_731311	7A2	41.36
	wPt_8473	7A2	42.93
	wPt_1179	7A2	42.93
	IWA556	7A2	47.13
	IWA557	7A2	47.13
	IWA3336	7A2	48.12
	wPt_1252	7A2	49.1
	wPt_4126	7A2	56.91
**Group 23**	wPt_5283	7B	0
	IWA1526	7B	0.61
	IWA783	7B	2.56
	IWA1181	7B	2.56
	IWA8007	7B	6.25
	IWA1089	7B	8.18
	IWA2894	7B	16.14
	IWA2893	7B	16.45
	Xgwm400a	7B	17.38
	IWA3915	7B	18.87
	IWA3965	7B	18.87

	IWA4966	7B	18.87
	IWA5390	7B	18.87
	IWA8138	7B	18.87
	IWA1315	7B	18.87
	Xwmc76	7B	18.87
	IWA1437	7B	19.18
	IWA1438	7B	19.18
	IWA4092	7B	19.18
	IWA8177	7B	19.18
	IWA3506	7B	20.69
	IWA7233	7B	20.69
	IWA3572	7B	21
	IWA7232	7B	21
	IWA3663	7B	29.67
	IWA1543	7B	29.67
	IWA5661	7B	29.67
	IWA6788	7B	29.67
	wPt_9467	7B	35.43
	IWA832	7B	36.92
	IWA1419	7B	36.92
	IWA4249	7B	36.92
	IWA306	7B	36.92
	IWA507	7B	36.92
	IWA2027	7B	36.92
	IWA3437	7B	36.92
	IWA3438	7B	36.92
	IWA3852	7B	36.92
	IWA3854	7B	36.92
	IWA4151	7B	36.92
	IWA4191	7B	37.21
	IWA3655	7B	37.21
	IWA4250	7B	37.21
	IWA6712	7B	37.21
	IWA7450	7B	37.21
	IWA8625	7B	37.21
	IWA5171	7B	37.21
	IWA1420	7B	37.21
	IWA3807	7B	37.21
	IWA4190	7B	37.21
	IWA8387	7B	37.21
	IWA4857	7B	39.92
	IWA8550	7B	39.92
	wPt_3833	7B	42.69
**Group 24**	IWA3749	7D1	0
	wPt_664286	7D1	1.01
	wPt_663849	7D1	1.31
	wPt_0303	7D1	3.21

	IWA4548	7D1	4.58
	Xbarc184	7D1	5.59
	wPt_798604	7D1	5.59
	Xgwm111	7D1	6.19
**Group 25**	wPt_665687	7D2	0
	wPt_664400	7D2	0.29
	Xgdm136	7D2	12.4
	IWA304	7D2	20.81
	IWA2273	7D2	25.04
	Xbarc172	7D2	26.9
	IWA6822	7D2	46.49
	IWA1323	7D2	46.81
	Vrn_D3	7D2	73.08
	Xgwm130	7D2	85.75
	wPt_733087	7D2	85.75
	wPt_744349	7D2	96.83
