# Supplementary material for: Heading Date QTL in Winter Wheat (Triticum aestivum L.) Coincide with Major Developmental Genes VERNALIZATION1 and PHOTOPERIOD1
Source: PLoS One. 2016 May 10;11(5):e0154242. doi: 10.1371/journal.pone.0154242 (PMC4862677; doi:10.1371/journal.pone.0154242)
Supplement: S4 Table — (PDF) [file pone.0154242.s007.pdf]

S4 Table. Distribution of markers on linkage map of the AGS 2000 by 26R61 RIL mapping population.

Chromosome	Length (cM)	Number of Unique loci	Number of markers
1A	147.58	107	229
2A	246.33	106	169
3A	179.88	50	101
4A	162.79	66	115
5A1	18.7	14	14
5A2	173	65	85
6A	210.27	99	164
7A1	90.81	45	68
7A2	56.91	24	47
A-Genome	1286.27	576	992
1B	96.13	65	115
2B	275.13	180	533
3B	220.6	136	216
4B	94.99	57	71
5B	283.78	141	224
6B	151.54	76	201
7B	42.69	23	54
B-Genome	1164.86	678	1414
1D1	59.5	18	55
1D2	58.63	18	27
2D	88.52	21	26
3D	37.66	14	35
4D	13.35	3	3
5D	175.56	33	41
6D	136.99	24	28
7D1	6.19	8	8
7D2	96.83	12	12
D-Genome	673.23	151	235
Total	3124.26	1405	2641

Summary

Marker type	Number of Unique loci	Number of markers
iSelect SNP	791	1724
DArT	419	720
SSR	188	190
KASP	5	5
STS	2	2
