# Supplementary material for: Heading Date QTL in Winter Wheat (Triticum aestivum L.) Coincide with Major Developmental Genes VERNALIZATION1 and PHOTOPERIOD1
Source: PLoS One. 2016 May 10;11(5):e0154242. doi: 10.1371/journal.pone.0154242 (PMC4862677; doi:10.1371/journal.pone.0154242)
Supplement: S6 Table — Days to heading were evaluated in the greenhouse following 8 weeks (8W), 4 weeks (4W) and 2 weeks (2W) of vernalization treatment. Heading date was recorded as day of year when lines were grown in the field in Baton Rouge, LA, Plains, GA and Raleigh, NC during 2012, 2013 and 2014. (PDF) [file pone.0154242.s009.pdf]

S6 Table. Heading date of AGS 2000, 26R61 and the RILs in the greenhouse and field. Days to heading were evaluated in the greenhouse following 8 weeks (8W), 4 weeks (4W) and 2 weeks (2W) of vernalization treatment. Heading date was recorded as day of year when lines were grown in the field in Baton Rouge, LA, Plains, GA and Raleigh, NC during 2012, 2013 and 2014.

	Days to Heading			Day of the Year				
Entry	8W	4W	2W	LA12	GA12	NC12	NC13	NC14
26R61	51	77	113	77	82	91	108	116
AGS_2000	52	79	104	75	80	89	106	115
Ril_1	56	84	99	74	82	90	108	116
Ril_2	60	82	123	80	85	91	107	118
Ril_3	57	81	124	81	81	91	107	116
Ril_4	58	67	97	68	75	85	107	114
Ril_5	56	70	111	67	77	84	107	113
Ril_6	67	79	108	72	80	89	106	117
Ril_7	60	109	132	.	.	.	108	116
Ril_8	68	66	98	69	74	83	105	114
Ril_9	61	94	99	70	79	86	108	116
Ril_10	62	76	98	73	80	90	106	117
Ril_11	57	65	101	69	78	89	106	116
Ril_12	58	69	99	72	77	87	106	114
Ril_13	56	73	106	72	81	89	109	116
Ril_14	60	84	107	74	80	92	109	116
Ril_15	64	76	98	73	79	90	105	117
Ril_16	57	67	98	68	75	85	105	114
Ril_17	59	78	115	75	79	88	106	114
Ril_18	87	120	130	95	98	116	123	121
Ril_19	57	73	94	67	76	85	108	114
Ril_20	56	79	112	76	81	90	106	116
Ril_21	68	80	116	75	82	92	110	116
Ril_22	59	78	102	75	82	93	107	116
Ril_23	55	76	123	69	77	88	110	116
Ril_24	50	75	118	76	80	85	107	114
Ril_25	53	79	117	74	82	88	106	116
Ril_26	60	81	107	74	80	90	108	116
Ril_27	58	69	95	68	76	84	106	112
Ril_28	60	72	101	73	78	85	108	115
Ril_29	51	76	107	83	86	95	111	120
Ril_30	62	73	113	81	86	93	109	119
Ril_31	54	76	111	70	76	83	107	114
Ril_32	66	92	106	79	82	92	108	117
Ril_33	54	74	100	74	78	85	107	116
Ril_34	52	84	127	84	85	93	111	118
Ril_35	54	78	102	72	80	91	110	117
Ril_36	60	103	134	80	83	91	115	121
Ril_37	50	77	112	69	74	84	108	113

Ril_38	55	76	114	77	81	91	108	116
Ril_39	52	74	116	68	79	86	105	114
Ril_40	59	76	120	79	83	91	112	117
Ril_41	53	73	117	74	80	86	109	117
Ril_43	48	70	96	69	77	85	109	116
Ril_44	61	78	125	75	79	87	106	113
Ril_45	68	78	117	77	79	86	106	112
Ril_46	52	74	112	77	79	85	105	114
Ril_47	55	70	104	70	80	87	108	117
Ril_48	55	63	97	71	77	86	109	114
Ril_49	54	76	110	78	82	92	108	118
Ril_50	65	86	109	73	80	87	110	120
Ril_51	64	77	124	76	81	88	106	116
Ril_52	54	78	119	75	81	89	108	117
Ril_53	68	67	102	73	81	89	109	117
Ril_54	59	100	136	90	86	96	112	117
Ril_55	65	90	119	72	81	90	109	117
Ril_56	55	74	99	70	79	84	104	114
Ril_57	59	67	102	77	83	96	112	118
Ril_58	57	74	108	75	81	89	106	113
Ril_59	50	69	113	69	76	85	104	111
Ril_60	55	65	107	76	82	91	108	117
Ril_61	62	76	99	75	82	90	111	118
Ril_62	55	71	104	69	77	86	107	115
Ril_63	56	80	113	77	82	89	106	116
Ril_64	56	80	112	78	83	90	107	115
Ril_65	63	73	112	74	80	87	106	116
Ril_66	65	82	122	95	96	106	121	126
Ril_67	49	79	123	71	77	86	106	114
Ril_68	54	95	129	76	81	88	111	118
Ril_69	63	71	110	72	81	90	111	117
Ril_70	52	78	114	75	79	88	106	111
Ril_71	58	82	93	71	78	88	107	116
Ril_72	59	80	110	76	79	90	108	118
Ril_73	51	64	103	68	75	84	107	112
Ril_74	55	72	110	74	79	85	105	115
Ril_75	60	86	126	.	.	87	107	116
Ril_76	56	86	109	75	81	91	108	118
Ril_77	73	87	106	.	95	108	121	124
Ril_78	55	82	99	70	77	86	105	115
Ril_79	47	69	101	69	76	84	106	114
Ril_80	59	81	119	80	81	89	107	118
Ril_81	58	66	90	69	79	85	109	116
Ril_82	57	82	117	80	81	88	107	116
Ril_83	57	74	104	71	79	87	107	117
Ril_84	52	75	128	75	81	89	107	118
Ril_85	58	71	101	69	79	87	108	116

Ril_86	55	84	131	86	84	91	107	117
Ril_87	60	77	108	68	77	89	107	124
Ril_88	54	75	107	69	79	87	108	114
Ril_89	73	81	107	.	94	105	122	122
Ril_90	86	92	112	77	83	92	111	117
Ril_91	62	83	106	78	82	91	108	115
Ril_92	84	80	101	.	95	107	118	122
Ril_93	58	84	110	71	79	91	106	116
Ril_94	63	74	122	77	80	90	107	116
Ril_95	62	80	113	78	82	92	109	118
Ril_96	54	72	112	71	77	84	105	112
Ril_97	56	77	121	77	82	90	106	116
Ril_98	60	104	102	67	78	86	107	117
Ril_99	59	76	118	74	79	87	106	116
Ril_100	56	80	97	69	78	88	105	116
Ril_101	59	80	115	78	82	91	107	116
Ril_102	55	77	116	77	80	89	105	116
Ril_103	56	84	102	68	78	88	107	115
Ril_104	58	81	99	74	80	90	108	117
Ril_105	60	77	110	71	81	83	104	112
Ril_106	51	74	106	68	76	86	108	117
Ril_107	63	83	108	72	78	86	106	116
Ril_108	56	72	100	72	80	88	106	117
Ril_109	51	71	111	74	81	90	108	120
Ril_110	55	82	127	82	83	93	107	119
Ril_111	54	74	129	76	82	89	109	117
Ril_112	54	73	116	.	.	.	105	116
Ril_113	54	79	117	78	83	92	112	117
Ril_114	62	86	97	.	.	.	107	116
Ril_115	56	87	98	76	83	92	107	118
Ril_116	51	79	113	71	79	89	109	116
Ril_117	49	78	87	70	78	89	107	116
Ril_118	48	66	91	68	78	84	107	116
Ril_119	54	81	126	.	.	.	107	117
Ril_120	66	85	105	95	96	111	120	121
Ril_121	52	74	108	77	83	91	108	118
Ril_122	49	82	103	71	79	91	107	117
Ril_123	52	68	105	74	79	86	107	116
Ril_124	61	96	124	80	85	92	110	121
Ril_125	52	80	126	76	81	87	107	116
Ril_126	55	87	116	95	93	107	116	121
Ril_127	59	80	103	77	82	91	108	117
Ril_128	61	93	115	82	87	94	114	119
Ril_129	58	89	123	73	81	92	112	119
Ril_130	52	71	96	69	76	83	108	114
Ril_131	62	79	103	70	79	89	108	115
Ril_132	59	88	103	78	85	95	114	122

Ril_133	54	81	106	81	84	94	112	117
Ril_134	56	130	140	.	.	.	116	119
Ril_135	54	129	142	95	96	95	112	119
Ril_136	55	82	103	74	79	89	106	116
Ril_137	57	87	123	78	88	92	112	117
Ril_138	52	82	98	72	80	90	109	118
Ril_139	54	108	119	72	79	90	107	117
Ril_140	50	85	131	77	.	86	105	116
Ril_141	53	83	106	76	82	89	105	116
Ril_142	63	80	103	.	95	111	123	114
Ril_143	52	84	97	68	77	85	105	115
Ril_144	50	81	135	75	81	90	110	118
Ril_145	61	92	112	82	90	98	111	119
Ril_146	58	90	108	83	86	97	112	116
Ril_147	56	86	107	78	82	90	110	121
Ril_148	55	83	111	80	83	92	109	116
Ril_150	50	86	101	69	79	86	104	116
Ril_151	77	137	142	87	88	92	119	121
Ril_152	62	84	117	80	83	93	113	118
Ril_153	53	87	115	95	98	99	108	113
Ril_154	51	84	107	79	84	95	106	114
Ril_155	55	90	111	71	79	88	113	116
Ril_156	63	118	140	71	.	90	111	118
Ril_157	65	104	113	95	97	111	122	121
Ril_158	53	86	131	77	82	90	107	117
Ril_159	53	123	141	84	98	101	117	122
Ril_160	62	73	112	74	81	91	108	117
Ril_161	65	87	117	95	98	111	122	117
Ril_162	60	82	130	95	96	109	122	124
Ril_163	67	90	121	95	98	108	124	118
Ril_164	49	84	106	70	78	88	106	115
Ril_165	50	131	144	95	95	98	114	119
Ril_166	61	123	142	95	98	100	117	119
Ril_167	65	87	100	78	84	94	112	121
Ril_168	49	67	106	74	79	87	106	117
Ril_169	53	123	137	95	96	98	111	117
Ril_170	53	91	119	87	89	94	109	116
Ril_171	67	131	139	95	98	116	123	126
Ril_173	59	136	143	95	98	103	117	122
Ril_174	52	64	99	69	79	88	111	114
Ril_175	55	129	137	95	98	99	114	121
Ril_176	49	69	97	69	76	84	106	115
Ril_177	55	120	143	95	98	99	110	118
Ril_178	55	87	124	78	86	93	114	120
